# Selected Monocyclic Monoterpenes and Their Derivatives as Effective Anticancer Therapeutic Agents

**DOI:** 10.3390/ijms22094763

**Published:** 2021-04-30

**Authors:** Mariola Zielińska-Błajet, Przemysław Pietrusiak, Joanna Feder-Kubis

**Affiliations:** Faculty of Chemistry, Wrocław University of Science and Technology, Wybrzeże Wyspiańskiego 27, 50-370 Wrocław, Poland; 234014@student.pwr.edu.pl

**Keywords:** carvone, carvacrol, perillyl alcohol, perillaldehyde, menthol, limonene, deep eutectic solvents, biological activity, antitumor agents, chemopreventive agents

## Abstract

Terpenes—a diverse group of secondary metabolites—constitute the largest class of natural products abundant in almost every plant species. The properties of concrete terpenes and essential oils have been intensively studied due to their widespread use in the pharmaceutical, food and cosmetics industries. Despite the popularity of these aromatic compounds, their derivatives, terpenoids, are still not comprehensively characterized despite exhibiting potent bioactive properties. This review aims to assess the anticancer properties of selected monoterpenes including carvone, carvacrol, perillyl alcohol, perillaldehyde, limonene, menthol and their derivatives while also evaluating potential applications as novel anticancer treatments. Special attention is paid to functional groups that improve the bioactivity of monoterpene molecules. This review also covers the therapeutic potential of deep eutectic solvents that contain monoterpene substances. Taken together, the literature supports the use of monoterpene derivatives in the development of new alternatives for disease treatment and prevention.

## 1. Introduction

Natural products derived from terrestrial plants, microorganisms, fungi and also marine organisms have long been sources of medicinal products that have since been exploited in medicine, pharmacology and biology [1]. Terpenes, also referred to as terpenoids or isoprenoids, represent the largest class of natural products with more than 88,000 structurally diverse compounds [2]. These substances are part of the secondary metabolism of vegetal and animal species. Structurally, terpenes are derived from isoprene units (C_5_H_8_). Depending on the number of linked isoprene units these chemical compounds are classified as hemi-, mono-, sesqui, -di-, sester-, tri-, sesquar-, tetra- (C_5_, C_10_, C_15_, C_20_, C_25_, C_30_, C_35_ and C_40_) and also polyterpenes. Many of these compounds have been extensively characterized and applied in the pharmaceutical, food, perfumery and cosmetic industries [2,3]. The broad range of the biological properties of terpenoids and their derivatives—including cancer chemopreventive, anti-inflammatory, antihyperglycemic, antibacterial, antifungal, antiviral, analgesic, antioxidant and anti-parasitic activities—have led to their widespread clinical application [4].

**Figure 1:**
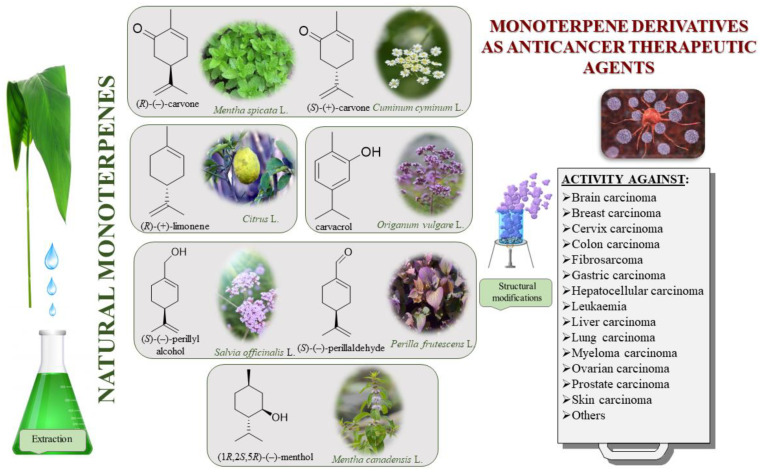
Division of various monoterpenes and their antitumor activity.

The bioactivities of monoterpenes are extensively reviewed in the literature. Many of these describe biomedical applications of essential oils and unmodified terpenes [5,6,7,8,9,10,11,12]; in contrast, articles dedicated to the biological activity of monoterpene derivatives are particularly rare [3,13]. Modifications of these natural compounds with proven biological properties are highly desirable. Terpenes can be functionalized by adding an element with confirmed bioactivity or, more simply, introducing a simple group (e.g., ether, ester and epoxide) that, by creating one or more heteroatoms, diametrically changes the activity of said molecule. Given the increasing amount of monoterpene derivatives described in the literature, a review of recent developments on monoterpene derivatives and their specific biological applications is warranted. As such, this work focuses on the clinical potential of promising monocyclic monoterpenes and their derivatives as antitumor agents (Figure 1). Specifically, we explore the following monoterpenes: carvone, carvacrol, perillyl alcohol, perillaldehyde, limonene and menthol.

Cancer is a complex, multifactorial disease involving uncontrolled cell growth that can affect nearly every tissue type in the body. Given that its prevalence continues to grow at an unmatched rate, cancer now represents one of the most pressing public health concerns worldwide. Though synthetic anticancer compounds still occupy the largest sector of the current cancer treatment market, more than two-thirds of the drugs currently used clinically are either derived directly from natural products or developed based on their bioactivity [10,14]. The study of natural products has been the single most successful strategy for the discovery of new medicines used as anticancer therapeutic; therefore, in the present review, we systematize the subject of the activity of six selected naturally occurring monoterpenes and their derivatives in anticancer treatment.

We also describe in detail various examples of therapeutic deep eutectic solvents (THEDESs) that contain a monoterpene as one of its components. To the best of our knowledge, this is the first review of natural monoterpenes and their derivatives to emphasize their eutectic equivalents for the treatment of cancer, which is surprising given how many publications explore the biological applications of deep eutectic solvents (DES) [15,16,17]. We consider THEDESs integral among terpene applications due to the proven capacity of these systems to increase the bioavailability of the active pharmaceutical ingredients (APIs).

This review provides a large platform of monocyclic monoterpene derivatives possessing anticancer activity, but it offers also general access to bioactive compounds, the key to advancing biological and medical science. We queried the literature review on carvone, carvacrol, perillyl alcohol, perillaldehyde, limonene and menthol and focused on studies published in the last 10 years, though the individual description may refer to older manuscripts; we used many available literature resources, from both general databases (e.g., Scopus and Web of Science), as well as all internationally recognized scientific publishers.

## 2. Carvone and Its Derivatives

**Figure 2:**
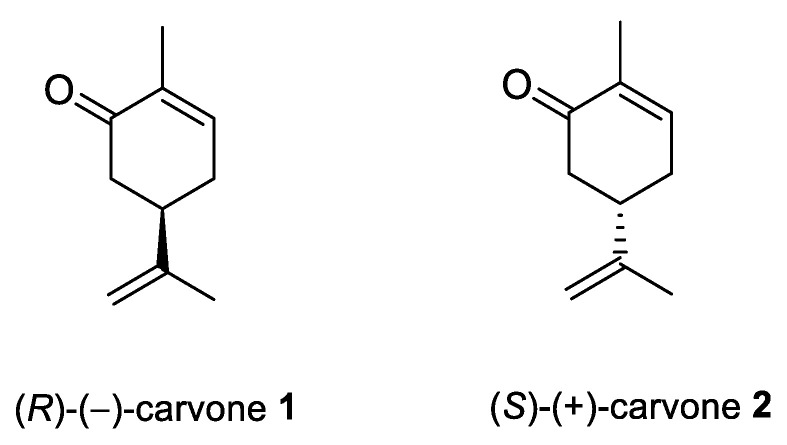
Stereoisomers of carvone.

Carvone (2-methyl-5-prop-1-en-2-ylcyclohex-2-en-1-one; 5-isopropyl-2-methyl-2-cyclohexen-1-one; CVN) is an important cyclic unsaturated monoterpenoid ketone which exhibits promising activities. This monoterpene is presents in large amounts in caraway, dill and spearmint essential oils [6,7]. This compound exists as two optical isomers that can perform different biological activities. (*S*)-(+)-Carvone is the main component of caraway (*Carum carvi* L.) and dill (*Anethum graveolens* L.) seed oils, with a scent similar to these herbs. In contrast, (*R*)-(−)-carvone occurs in spearmint oil (*Mentha spicata* L.), which is steam-distilled from the leaves of the plant and has a minty, sweet scent (Figure 2). Carvone has several established applications: in the food industry, for fragrance and flavor; in agriculture as a sprouting inhibitor; and in medicine, due to its anticonvulsant and antioxidant properties [18]. Moreover, it has antimicrobial effects against various microorganisms [7,19]. Current research supports carvone’s therapeutic application to brain, colon, skin, myeloma and prostate cancer [14].

Mesa-Arango et al. examined the cytotoxicity of CVN in vitro, using human cervix epithelioid carcinoma cells (HeLa cell line ATCC, CCL-2) and *Cercopithecus aethiops* African green monkey kidney cells (Vero cell line, ATCC CCL-81). CVN was cytotoxic to the HeLa tumor cell line (CC_50_ = 74.5 μg/mL) and had little effect on the Vero non-tumor cell line (CC_50_ > 200 μg/mL) (Table 1). Furthermore, the cytotoxic effects of CVN on HeLa cells were dose-dependent [20].

Zyad and co-workers evaluated the antitumor activity of carvone in vitro, using five tumor cell lines: P-815, K-562, CEM, MCF-7 and MCF-7 gem. They found that this monoterpene has an important effect, especially against the P-815, K-562 and CEM tumor cell lines with IC_50_ values ranging from 0.11 to 0.17 μM (Table 1) [21].

The anticancer potential of carvone was tested by Aydin et al. in cultured primary rat neurons and N2a neuroblastoma (NB) cells. The study revealed that high doses of CVN (≤ 100 mg/L) conferred a strong cytotoxic effect on healthy rat neurons and NB cells. The examinations also demonstrated weak antioxidant functions and little anticancer potentials in vitro. Studies suggest that CVN can be useful as a brain cancer chemopreventive and chemotherapeutic agent [22].

The biological effect of (*S*)-(+)- and (*R*)-(−)-carvone enantiomers on human colon tumor cells (HT29) and healthy colonic epithelial cells (CCD 841 CoTr) was evaluated by Paduch et al. [23]. The cytotoxicity and cell sensitivity of these stereoisomers were determined by using NR and MTT assays. The finding suggest that the divergent activity of enantiomers depends on the assay method used. (*R*)-(−)-Carvone demonstrated less toxic activity in tumor and healthy cells compared with its (*S)*-(+) enantiomer in the NR assay. (+)-Carvone was 59.4% and 27.1% more cytotoxic to tumor and healthy cells, respectively, than its (−)-stereoisomer. Tumor cells were much more sensitive to (*R*)-(−)-carvone whereas healthy cells were a little more susceptible to (*S*)-(+)-enantiomer in the MTT method. Both isomers were less cytotoxic to healthy cells (IC_50_ = 475 and 310 μg/mL, respectively). Similar results were obtained for this pair of enantiomers in all of the cancer lines (OVCAR-8, HCT-116 and SF-295) tested (Table 1) [24].

Another group of scientists, Nalini and co-workers [25] examined the chemopreventive potential of (*S*)-(+)-carvone against colon carcinogenesis. The considerable inhibitory effects of (+)-CVN on 1,2-dimethylhydrazine (DMH)-induced colon carcinogenesis was observed. The authors suggest that this effect could be related to the pro-oxidant–antioxidant balance, carcinogen detoxification and also its anti-proliferative effects. A dose of 10 mg/kg body weight prevented chemically induced colon carcinogenesis probably by reducing the neoplastic and inflammatory responses induced by DMH. 

(*S*)-(+)-Carvone also efficaciously protected against DMBA induced skin carcinogenesis by modulating the activities of phase I and phase II detoxification enzymes and inducing cancer cell apoptosis. Oral administration of (+)-CVN restored the molecules involved in the apoptosis process [26].

Ding and Chen [27] demonstrated a significant anticancer effect on the myeloma cancer cells of CVN in a dose-dependent manner (IC_50_ = 20 μM against KMS-5 myeloma cells) (Table 1). The antiproliferative effect was related to induction of apoptosis and G2/M cell cycle arrest. CVN could inhibit cell invasion and the expression of the p-P38 protein at this IC_50_.

Studies conducted by Wang et al. [28] showed that (*S*)-(+)-CVN significantly inhibited cell proliferation and induced apoptosis in a DMBA-induced tumor hamster model of oral mucosal carcinogenesis. 

**Figure 3:**
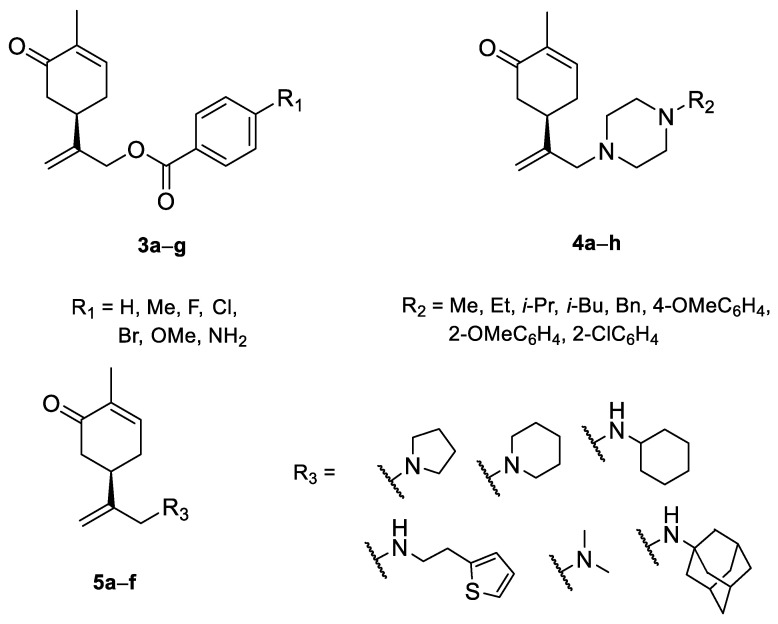
Carvone derivatives with anticancer activities.

A series of carvone derivatives with lipophilic benzoates and hydrophilic amines linked to the terpenoid moiety were synthesized by Dong and co-workers (Figure 3) [29].

All of the prepared compounds including (*R*)-(−)-carvone (L-carvone) were screened in vitro for their antiproliferative activities against human prostate (LNCaP) cancer cells, using MTT assay (Table 1). It was found that the presence of 4-methylbenzoyl **3b**, 4-methoxybenzoyl (**3f**) and 4-aminobenzoyl groups (**3g**) in CVN notably increased the antiproliferative activity compared with non-substituted (−)-carvone. The incorporation of *N*-alkylpiperazine or *N*-benzylpiperazine into the carvone moiety (**4a**–**e**) did not increase the antiproliferative effect of (−)-CVN noticeably. However, the presence of *N*-arylpiperazine scaffold in the structure of (*R*)-(−)-carvone (**4f**–**h**) significantly increased the antiproliferative activity of (−)-CVN. Amine substituted compounds **5a**–**f** did not improve the antiproliferative properties except for compound **5d** with a 2-thiopheneethylamine group (2-methyl-5-{1-[(2-thiophen-2-ylethylamino)methyl]vinyl}cyclohex-2-enone). The cell growth inhibitory effect of these compounds correlates with ERK activation and p21^waf1^ induction.

**Figure 4:**
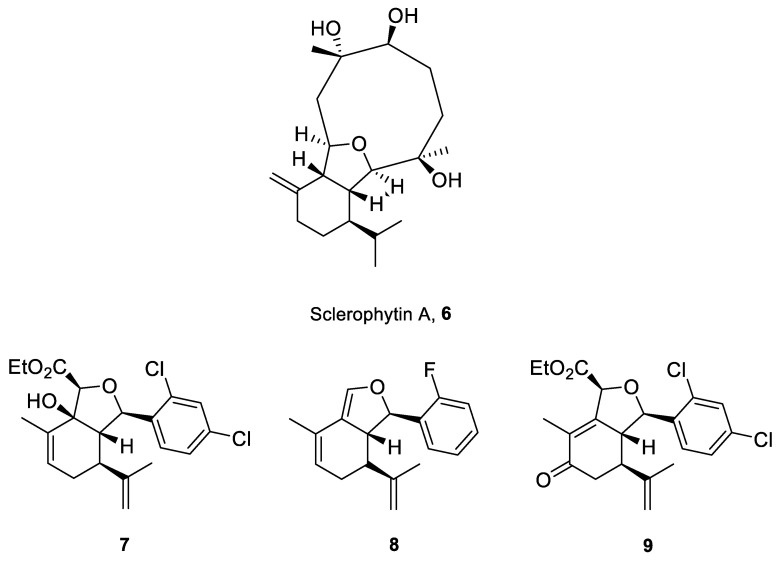
The most active analogues of sclerophytin A **6** exhibited anticancer activity.

Structurally related sets of (*S*)-(+)-carvone-based hydroisobenzofuran analogues of sclerophytin A **6** were synthesized by Chambers and co-workers via an aldol-cycloaldol sequence (Figure 4) [30]. 

These compounds were screened for their antitumor activity against the human epithelial carcinoma cell line (KB-3), using the MTT assay. Among the tested classes of the compound, promising results were obtained for alcohol **7**, diene **8** and enone **9** (Figure 4). Carvone derivative **9** showed significant differential activity against RPMI-8226 leukemia and the prostate cancer cell lines(PC-3). The results indicated that the most active was diene **8**, having considerably varied activity against the entire leukemia panel and the non-small lung cancer line. A subsequent five-dose testing of **8** revealed GI_50_ = 0.148 μM and LC_50_ = 9.36 μM for RPMI-8226 leukemia cell line and GI_50_ = 0.552 μM and LC_50_ = 26.8 μM for the HOP-92 non-small-cell lung cancer line (Table 1).

**Figure 5:**
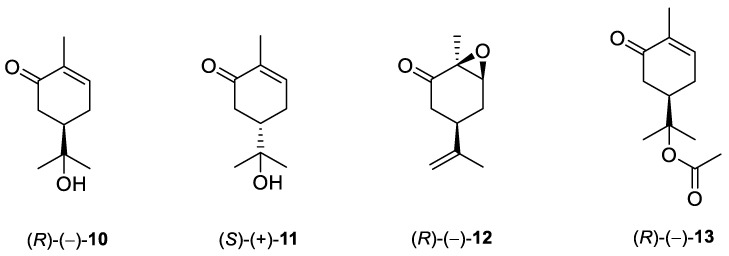
Carvone derivatives examined against tumor cell lines: HCT-116 (colon), OVCAR-8 (ovarian) and SF-295 (brain).

De Sousa and co-workers selected eighteen monoterpenes including carvone derivatives **10**–**13** and evaluated their cytotoxic activity against tumor cell lines: HCT-116 (colon), OVCAR-8 (ovarian) and SF-295 (brain), using the MTT assay to establish the corresponding structure–activity relationships (SARs) (Figure 5) [24]. 

The authors examined the influence of hydroxyl and epoxide groups and ester function in the structure of (*R*)-(−)- and (*S*)-(+)-carvone enantiomers in terms of cytotoxic activity. Only the hydroxylation of a double bond in (−)-carvone significantly enhanced its anti-proliferative effect. (−)-8-Hydroxycarvotanacetone **10** was the most cytotoxic compound (GI = 61.59–94.01% at the concentration of 25 μg/mL) among the carvone derivatives tested (Table 1).

**Figure 6:**
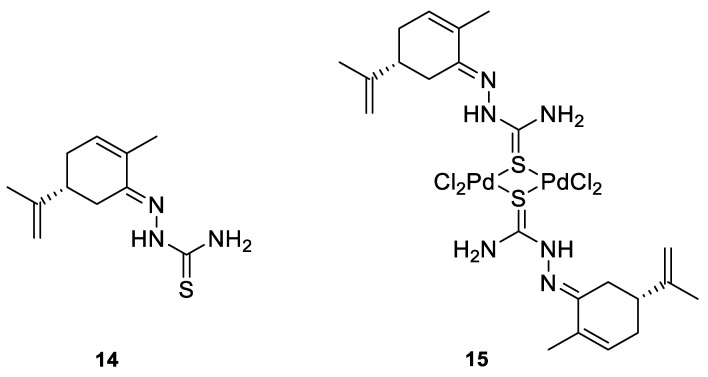
(*R*)-(−)-Carvone thiosemicarbazone **14** (L^1^) and Pd(II) complex **15** (PdL^1^Cl_2_).

Many studies have demonstrated that metal complexes with thiosemicarbazones exhibit also anticancer activity [31]. Recently synthesized copper(II) complexes with various thiosemicarbazones are highly potent against HepG-2 (human liver hepatocellular carcinoma), NCI-H460 (human large-cell lung carcinoma) and HeLa (human cervical carcinoma) cells with significantly lower IC_50_ values than cis-platin [32]. Kokina et al. prepared Cu(I) and Pd(II) complexes with chiral thiosemicarbazones containing fragments of (−)-carvone and determined the cytotoxicity of the ligand L^1^ (**14**) and the complex PdL^1^Cl_2_ (**15)** for the adenocarcinoma Hep2 cell line (Figure 6) [33]. The results showed that complex **15** was more cytotoxic than thiosemicarbazone **14** (Table 1).

In summary, carvone and two carvone enantiomers decreased the viability of cancer cells. The (+)-carvone and the opposite (−)-isomer expressed diverse activity against healthy and tumor cells, depending on the cytotoxicity measurement method used (MTT or NR assay). Both isomers induced less cytotoxicity against healthy cells. Furthermore, this study demonstrated that carvone derivatives, in many cases, exhibit more potent cytotoxic effects on certain cancer cells compared with the starting terpene. The insertion of, for example, a hydroxyl or 4-substituted benzoyl groups as well *N*-arylpiperazine scaffold in the structure of (*R*)-(−)-carvone significantly increases the antiproliferative capacity of (−)-CVN.

## 3. Limonene and Its Derivatives

**Figure 7:**
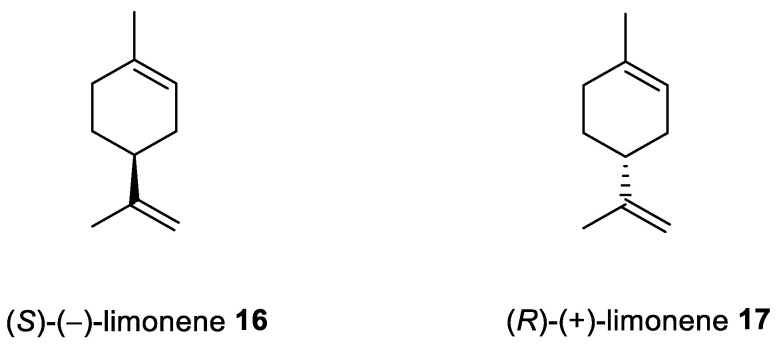
Stereoisomers of limonene.

Limonene [*p*-mentha-1,8-diene, carvene, 4-isopropenyl-1-methyl-1-cyclohexene] is a 10-carbon cyclohexanoid monoterpene derivative found in lemon, orange and grapefruit essential oils (Figure 7). Numerous therapeutic benefits (e.g., anti-inflammation, antitumor and anti-asthma) have been attributed to limonene [34,35]. Moreover, this monoterpene is largely used in alimentary items, cleaning products and cosmetic formulations where it is the most frequent and desirable of the fragrances used.

Many reviews [36,37] have been published with in-depth descriptions of the latest developments related to the therapeutic effects of limonene. Its potent activity renders it a promising target in anti-inflammatory, antioxidant, antinociceptive, anticancer, antidiabetic, antihyperalgesic, antiviral and gastroprotective research. The anticancer properties of limonene have been assessed in various types of cancer inter alia as lung, prostate, breast, gastric, bladder and colon (Table 1). The antitumor properties of this monoterpene might be derived from its ability to trigger apoptosis and regulate the cell cycle [37].

**Figure 8:**
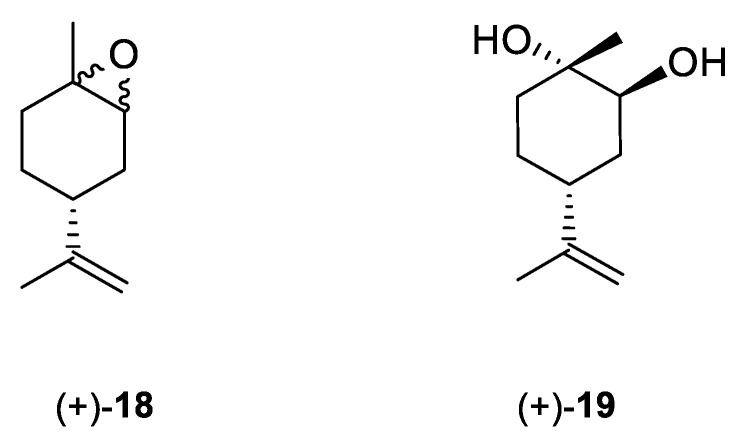
Structures of limonene derivatives: (+)-limonene 1,2-epoxide **18** and (+)-limonene-1,2-diol **19**.

Andrade et al. [24] investigated the cytotoxic potential of *p*-menthane derivatives, including (+)-limonene 1,2-epoxide (Figure 8, compound **18**). The cytotoxic activity of various monoterpene derivatives was evaluated in several human tumor cell lines: HCT-116 (colon), OVCAR-8 (ovarian) and SF-295 (brain). The results were assessed by using an intensity scale [38]: samples with weak cytotoxic activity have GI less than 50%, those with intermediate activity have GI between 51 and 75% and those with high activity—higher than 75%. The results showed that the tested epoxide derivative of (+)-limonene **18** exhibited intermediate to high GI activity (58.48–93.10%), specifically GI = 73.13% for the HCT-116 cell line, 93.1% for OVCAR-8 and 58.48% for SF-295 (Table 1). Interestingly, comparing (+)-limonene 1,2-epoxide **18** with (−)-carvone epoxide revealed the endocyclic ketone function does not contribute to the high cytotoxicity, as compound **18** was approximately 2.5- to 11-fold more cytotoxic than the (−)-carvone derivative.

Souto et al. [39] developed a solid lipid nanoparticles (SLNs) formulation with (+)-limonene 1,2-epoxide **18** and glycerol monostearate (LIM–SLNs) to evaluate the role of SLNs in lipid peroxidation and cytotoxicity, using a keratinocyte cell line derived from adult human skin (HaCaT). Loading the (+)-limonene derivative **18** into the glycerol monostearate SLNs significantly improved both lipid peroxidation and cytotoxicity in the HaCaT cell line. Moreover, the authors proposed a promising set of antioxidant and antitumor formulations. The cytotoxicity assay performed for blank SLNs in the HaCaT cell line showed cell viability above 82.41 ± 0.93% after 48 h at the highest concentration of 10 µg/mL (Table 1). The loading of (+)-limonene 1,2-epoxide **18** into SLNs decreases cell viability to 76.27 ± 1.63% (using the same concentration and incubation time) while remaining above the 70%required to confirm a non-cytotoxic profile. When we compare the results from Andrade et al. [24] in which tumor cell lines were used, with Souto et al. work [39], the growth inhibition was below 30%, which might indicate a low toxic effect on the non-tumor cell line HaCaT. Therefore, when the cytotoxicity profile becomes too high for terpenes with highly effective anticancer activity, one should consider the appropriate formulation type of such an active substance, e.g., loading active terpene compounds into an SLNs system.

Another derivative of this monoterpene monocyclic compound is its diol **19** (Figure 8) [40]. Lee et al. tested the therapeutic effects of *P. koraiensis* pinecone water extract on human lung adenocarcinoma cells. Additionally, they isolated and identified the main molecular components of the pinecone extract, one of which was (+)-(1*S*,2*S*,4*R*)-limonene-1,2-diol **19.** The water extract of *P. koraiensis* pinecones exhibits cytotoxic activity, with an IC_50_ values ranging from 0.62 to 1.73 mg/mL in the A549, H1264, H1299 and Calu-6 human lung cancer cell lines by inducing apoptotic cell death in a caspase-3-dependent manner (Table 1). The authors concluded that the cytotoxicity of *P. koraiensis* pinecone water extract is likely related to the properties conferred by its individual substances [41].

In summary, limonene derivatives that are slightly structurally modified are promising candidates to combat antitumor diseases; most notably, (+)-limonene 1,2-epoxide (**18**) and (+)-limonene-1,2-diol (**19**). Considering the enormous antitumor potential of pure, unsubstituted limonene, further research is warranted on other structures and modifications, for example, incorporating an *N*-alkylpiperazine or *N*-arylpiperazine scaffold, ester bridge, etc.

## 4. Perillyl Alcohol and Its Derivatives

Perillyl alcohol ([4-(prop-1-en-2-yl)cyclohex-1-en-1-yl]methanol; POH) is a naturally occurring monocyclic terpene obtained from limonene and the mevalonate pathway in some plants, e.g., lavender, spearmint and peppermint. POH especially appears in the essential oils of mints, cherries and citrus fruits [42]. POH is often used as an ingredient in cosmetics and cleaning agents and is known for its lack of toxicity. In recent years, POH and its derivatives have been thoroughly investigated and developed as a drug component of cancer treatment [43].

**Figure 9:**
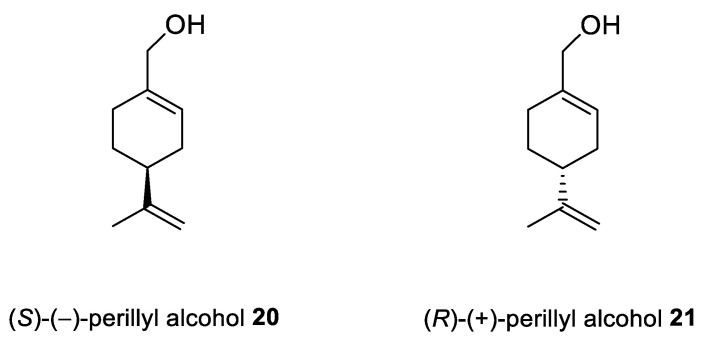
Stereoisomers of perillyl alcohol.

Chen et al. [44,45] pointed to POH (Figure 9) as an exception in cancer therapy. The natural compound **20** shows positive results after application through intranasal administration for glioma therapy. This drug delivery method enables reaching the brain directly by nose-to-brain transport, avoiding a blood–brain barrier (BBB). Intranasal application of NEO100 (clinical-grade **20**) as an antitumor agent represent the unique opportunity to deliver this type of drug through the cranial nerve. Moreover, this type of delivery minimizes side effects by reducing exposure through systemic circulation.

To overcome POH’s low solubility and high volatility issues, Rezende et al. studied (*S*)-(−)-perillyl alcohol **20** complexed with β-cyclodextrin (β-CD) as a possible chemotherapeutic treatment. In this research, POH/β-CD inclusion complexes in a molar proportion of 1:1 have been successfully prepared. Thermal analysis, Fourier-transform infrared spectroscopy (FTIR) and SEM confirmed the presence of POH enclosed in the β-CD cavity. The new complexes have been tested against human L929 fibroblasts. After 24 h of incubation, they did not see any signs of cytotoxicity against L929. The histopathological results showed that POH/β-CD treatment at a dose of 50 mg/kg improved the inhibition of tumor growth by about 60% in vivo on sarcoma S180-bearing mice (Table 1). Proliferation properties were tested in situ by immunostaining the Ki67 antigen, which revealed a substantially reduced population of cycling cells. The results obtained by Rezende and his co-workers support the potential benefits of using POH during tumor treatment and highlight the advantages of β-CD-docking as an improvement of the drug’s activity in vitro and in vivo along with decreasing the risk of volatility [46]. 

**Figure 10:**
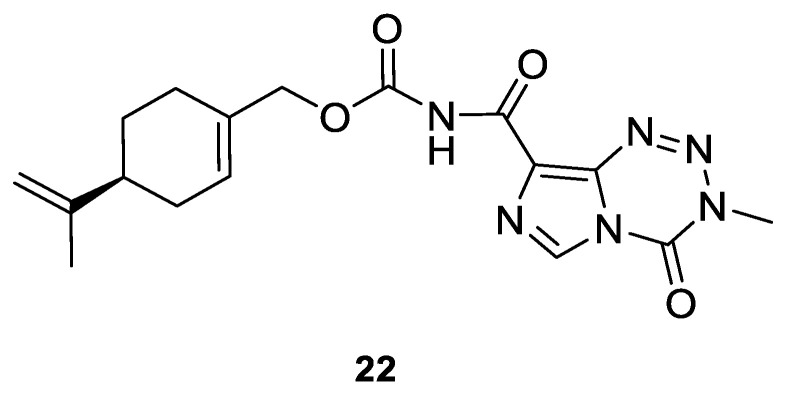
The structural formula of NEO212.

Silva-Hirschberg and co-workers explored the antitumor effects of NEO212, using an experimental compound composed of covalently conjugated (*S*)-(−)-POH **20** and temozolomide (TMZ) (Figure 10). NEO212 (**22**) was tested on *Mycosis fungoides* and in Sézary syndrome—lymphoma cell lines—in vitro. HUT-78, HUT-102 and MyLa cells were treated with NEO212 (**22)** and the effects on apoptosis and viability were characterized. Cells were exposed to the increasing concentrations (1 to 300 µM) of NEO212 (**22**). HUT-78 and HUT-102 cells appeared to be more sensitive to NEO212 (IC_50_ = 3–9 µM) than MyLa cells (IC_50_ = 85–130 µM) (Table 1). As a result of the binary composition of NEO212 (**22**), this compound is more cytotoxic than its individual components [47].

**Figure 11:**
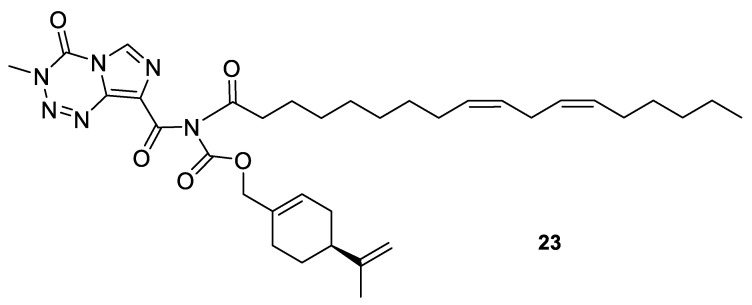
Chemical structure of NEO412 drug.

A novel agent, NEO412 (**23**) (Figure 11) was designed and studied in response to the need for improved transdermal treatment of melanoma. NEO412 (**23**) is a compound built by the covalent conjugation of three agents: TMZ, (−)-POH and linoleic acid. Swenson et al. investigated the anti-melanoma potency of this novel agent in vitro and in mouse models in vivo. The research shows that NEO412 (**23**) efficaciously killed melanoma cells in vitro. In vivo, NEO412 inhibited tumor growth when applied to the skin of animals bearing tumors (Table 1). This effect included a combination of increased tumor cell death with only minor blood vessel development. Drug-treated mice exhibited no apparent damage relative to healthy skin in response to periodic drug application [48].

**Figure 12:**
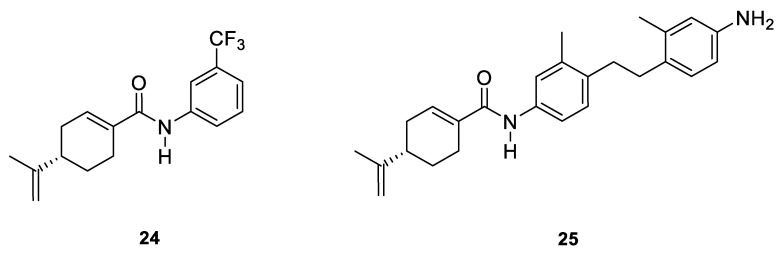
Selected *N*-arylamide derivatives of (*S*)-perillic acid.

Two novel amide derivatives **24** and **25** of (*S*)-perillic acid (Figure 12) were designed and synthesized. These new analogues caused a significant anti-proliferative effects in the HC and GBA cell lines. Compound **25** exhibited a magnified inhibition effect on U251 and HepG2 cell lines with an IC_50_ of 3.10 and 1.49 µg/mL, respectively. The research group investigated the in vivo anticancer activity and toxicity against organ or tissue in mice with inoculated hepatoma (H22) cancer cells. The results showed that analogues **24** and **25** exhibit inhibition properties against H22 cells with no major damage to tissues or organs (Table 1) [49]. 

**Figure 13:**
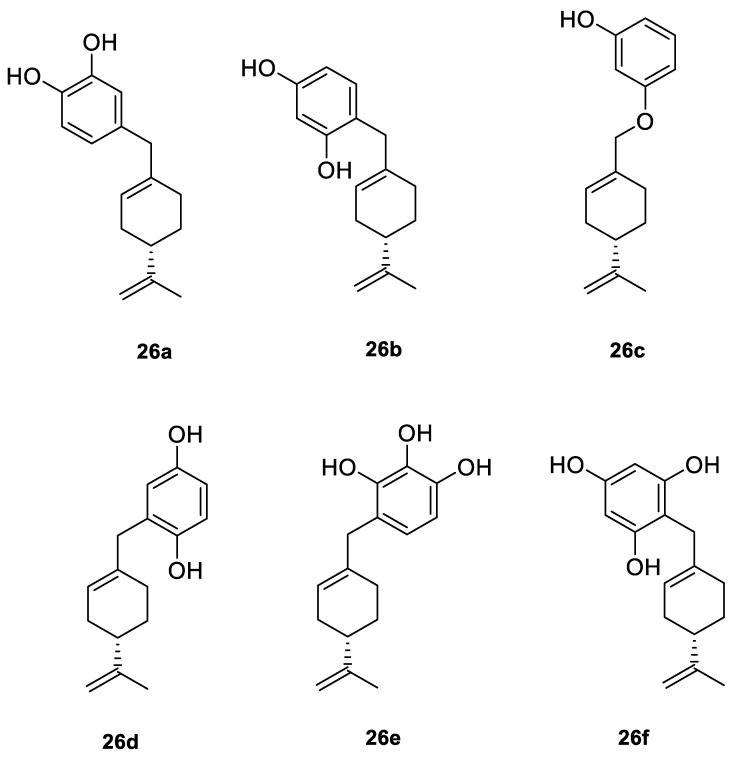
Selected cyclodiprenyl phenols derivatives.

A series of new cyclodiprenyl phenols **26a**–**f** (Figure 13) were synthesized from perillyl alcohol and synthetic phenols. These novel compounds were investigated as potential antiproliferative agents against a multitude of cancer cell lines. Meroterpenes were tested for their in vitro cytotoxicity in three human cancer cell lines: MCF-7 (breast cancer), PC-3 (prostate cancer) and HT-29 (colon cancer) and two human non-tumor cell lines: human dermal fibroblasts (HDFs) and colon epithelial cells (CoN) (Table 1). Among all of the new analogues tested, compound **26a** in particular exhibited strong antiproliferative activity against the breast and prostate cancer cell lines. In comparison, compounds **26b** and **26c** showed moderate activity. This research highlights the need for additional studies to support the therapeutical potential of these new derivatives [50].

## 5. Perillaldehyde and Its Derivatives

Perillaldehyde ((*S*)-4-(1-methylethenyl)-1-cyclohexene-1-carboxaldehyde; PAH) is a natural compound extracted from *Perilla frutescens* (L.) Britton. PAH is a monoterpenoid commonly used in perfumery due to its’ mint-like, cinnamon scent and also as a food additive. It exhibits antifungal, antidepressant, antioxidant and other clinically relevant properties. Most PAH investigations are based on the assumption that it has antitumor properties. Different derivatives of PAH with such properties have been designed, synthesized and investigated [51,52]. 

Zhang et al. [53] considered using PAH as an agent preventing gastric cancer growth and explored its molecular mechanism. In mouse and human gastric cancer cell lines, PAH induced activation of AMPK (AMP-activated protein kinase), responsible for the initiation of autophagy in different tissues, depending on time and concentration. The in vivo assays showed that a 4-week treatment with PAH in 100 mg/kg/day doses inhibited the growth of gastric cancer and fostered an increase in the concentration of autophagy-related proteins. PAH may be a drug incorporated into the treatment of gastric cancer in the near future.

**Figure 14:**
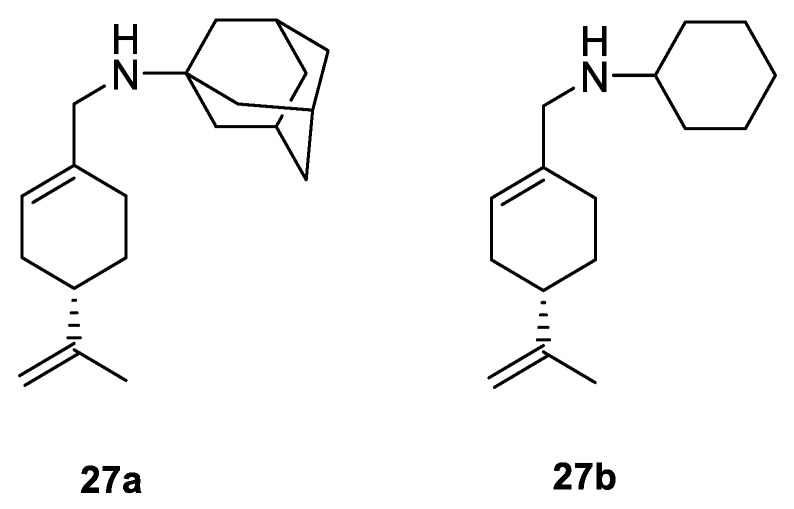
Chemical structure of most potent PAH derivatives.

A study by Hui and co-workers focused on the synthesis of several amino-modified derivatives of (*S*)-perillyl alcohol. These compounds were tested against human lung cancer A549 cells, human fibrosarcoma HT-1080 cells and human melanoma A375-S2 cells with POH as a comparison. Two of these derivatives **27a** and **27b** (Figure 14) were the most promising agents, due to their low IC_50_ below 100 µM (Table 1). This research shows that through its antiproliferative effects, compound **27a** was involved in the induction of apoptosis in human lung cancer cells [54].

**Figure 15:**
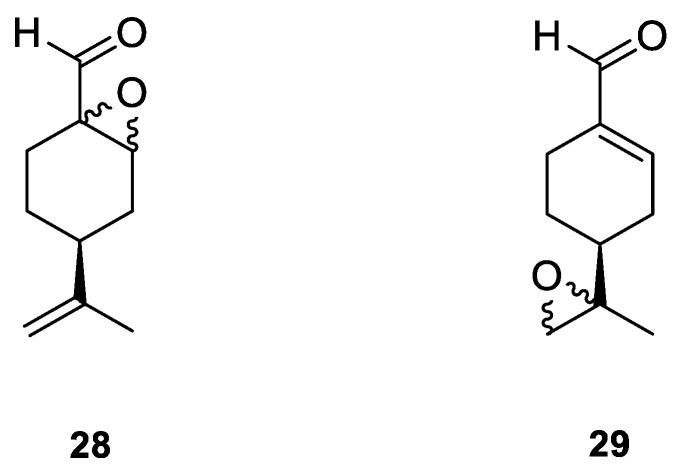
Selected PAH epoxide derivatives.

A novel agent, perillaldehyde 1,2-epoxide (PE) **28** (Figure 15) was studied to estimate its cytotoxicity and antitumor activity against a few human tumor cell lines. Colon carcinoma (HCT-116), ovarian cancer (OVCAR-8), leukemia (HL-60) and glioblastoma (SF-295) cell lines have been investigated. The application of **28** conferred over 95% cell growth inhibition with an IC_50_ = 9.70–23.61 µM against all types of human tumors cell lines assayed in this study (Table 1). The in vivo anticancer potential of PE (**28**) was assessed in sarcoma-bearing mice. Tumor growth inhibition rates were estimated at between 33 and 66% at two different doses. Histopathological analyses of the spleen, liver and kidney showed no morphological changes in mice dosed with PE. Hence, the authors concluded the PE (**28**) exhibits cytotoxic and anticancer properties via necrosis and apoptosis [55].

Andrade and co-workers [24] prepared several compounds occurring in essential oils of various groups of plants. This study was focused on investigating the cytotoxicity potential of *p*-menthane derivatives. One of its derivatives, (−)-perillaldehyde 8,9-epoxide **29** (Figure 15), generated the highest percentage of growth inhibition for ovarian adenocarcinoma, colon carcinoma and glioblastoma cells (GI = 96.32–99.89%) with an IC_50_ = 1.75–1.03 µL/mg at a concentration of 25 µg/mL. (−)-Perillaldehyde 8,9-epoxide induces apoptosis and necrosis, despite its high cytotoxicity, it is not hemolytic even at 500 µg/mL. The application of **29** to human leukemia cells inhibits the proliferation of these cells and induces apoptosis and necrosis (Table 1). Hence, (−)-perillaldehyde 8,9-epoxide is an attractive candidate for in vivo tests.

Another group of researchers loaded perillaldehyde 1,2-epoxide (**28**) into cationic solid lipid nanoparticles (cSLNs) with a monoclonal antibody for site-specific delivery to breast cancer cells. Solid lipid nanoparticles (SLNs) were selected for their biocompatible and biodegradable lipids compositions. This combination of components reduces the risk of cyto-genotoxic events. Nanoparticles may be produced containing cationic lipids and a positive charge can be tailored with a monoclonal antibody against human epithelial growth receptor [56].

In conclusion, POH and PAH have promising future in drug development. Much of the research has investigated the effects of structural modification, using many types of human cancer cell lines. Moreover, instead of the structural modification of POH and PAH, some studies concentrate on the delivery of unmodified monoterpenes to specifically target cancer cells [57]. This approach is a good prognostic for achieving greater success in delivering active compounds in accordance with the specific pharmacotherapeutic needs. It seems justified to assay POH and PAH derivatives in delivery systems such as nanoparticles, especially modified terpenes whose biological effectiveness is higher than their unsubstituted analogues.

## 6. Carvacrol and Its Derivatives

Carvacrol (2-methyl-5-(1-methylethyl)-phenol; CV, **30**) is a phenolic monoterpenoid, isomeric with thymol, found in essential oils of oregano (*Origanum vulgare* L.), thyme (*Thymus vulgaris*), pepperwort (*Lepidium flavum*), wild bergamot (*Citrus aurantium* var. *bergamia* Loisel.) and other plants. Carvacrol exhibits broad-spectrum bioactivity that may be useful in pharmacology, such as antimicrobial, antioxidant and anticancer activities [14,57,58]. Recently, the anticancer properties of CV have been described in preclinical models of breast, liver and lung carcinomas as acting on proapoptotic processes.

The effects of CV on breast cancer have been investigated in several studies. Arunasree [59] examined the mechanism of carvacrol-induced cell death in human metastatic breast cancer cells (MDA-MB 231). CV induced apoptosis occurred in a dose-dependent manner (IC_50_ = 100 μM) (Table 1). The author suggests that CV’s mechanism of action may be related to its antioxidant activity but is not associated with DNA damage. The data demonstrated the antitumor activity of carvacrol on metastatic breast cancer cells.

Baranauskaite et al. evaluated the anticancer activity of CV on human glioblastoma (U87) and triple-negative mammary gland adenocarcinoma breast cancer (MDA-MB231) cell lines in vitro. CV was about 1.5–2-fold more active against the MDA-MB231 cell line (*p* < 0.05) compared with the U87 cell line. It inhibited the growth of 50% of both tested cancer cell lines at concentrations of 199 μM (MD-MB231) to 322 μM (U87) (Table 1) [60].

The antitumor capacity of CV was also studied by using the 7,12-dimethylbenz[a]anthracene (DMBA) induced breast cancer model in female rats. Carvacrol had an antitumor effect independent of dose; at 100 mg/kg BW, CV treatment caused a significant decrease in the cumulative tumor volume down to 0.11 ± 0.05 cm^3^ compared with the 0.38 ± 0.04 cm^3^ average of the DMBA group (*p* < 0.001) and reduction to 0.44 cm^3^ of cumulative tumor volume compared with 6.10 cm^3^ of the DMBA group [61].

Recently, carvacrol was reported to affect breast cancer cells through TRPM7 mediated cell cycle regulation. The cell viability of breast cancer cell lines BT-483, BT-474, MCF-7, MDA-MB-231 and MDA-MB-453 was tested and TRPM7 was evaluated in MDA-MB-231, MCF-7 and HEK293 cells. The results showed that carvacrol inhibited the viability of breast cancer cells with different potencies. CV had the greatest effect on MDA-MB-231 and the least on MCF-7 at 200 μM. CV inhibited TRPM7 functions in MDA-MB-231, MCF-7 and HEK293 [62].

The carcinogenesis-reducing potential of CV was demonstrated by Ozkan and Erdogan [63]. These monoterpenes have dose-dependent antiproliferative effects on Hep G2 cells. The viability of the Hep G2 cells decreases when the cells are exposed to carvacrol at concentrations between 20 and 170 μg/mL and did not change at concentrations of ≥170 μg/mL (IC_50_ = 53.09 μg/mL) (Table 1). Likewise, the study by Zhuang and co-workers [64] indicated that carvacrol inhibits HepG2 cell growth by inducing apoptosis by direct activation of the mitochondrial pathway and the mitogen-activated protein kinase pathway. Importantly, selectivity was also observed with CV treatment: carvacrol induces stronger effects on HepG2 cells in the tested concentration ranges (0.05–0.4 mmol/L) compared with the healthy human fetal liver cells (LO2 cells). The treatment of LO2 cells with carvacrol (0–0.4 mmol L-1) for 24 h did not significantly affect cell viability (Table 1). Similarly, studies conducted by Elshafie et al. prove that the cell viability percentage of treated HepG2 by CV is significantly lower compared with healthy human renal HEK293 cells. The IC_50_ values for HepG2 are much lower than healthy renal cells, which supports the selectivity of carvone (Table 1) [65]. 

The effects of carvacrol on P-815 (murine mastocytoma), K-562 (human chronic myelogenous leukemia), CEM (acute T lymphoblastoid leukemia), MCF-7 (human breast adenocarcinoma) and its gemcitabine-resistant counterpart (MCF-7 gem) were evaluated by Zyad and collaborators [21]. They found that CV displays an antitumor activity against the P-815, K-562 and CEM tumor cell lines. The IC_50_ values ranged from 0.0067 to 0.042 μM (Table 1).

Jayakumar and co-workers [66] demonstrate that CV protects the antioxidant system in diethylnitrosamine (DEN)-induced hepatocellular carcinogenesis and also prevents lipid peroxidation and hepatic cell damage.

Another group investigated the effects of CV on proliferation, apoptosis and cell invasion, using human prostate cancer cells. They found that carvacrol negatively impacts cell viability, hampers invasion and induces apoptosis in DU145 cells via the mitogen-activated protein kinase (MAPK) signaling pathway [67]. Similar effects were reported by Khan et al. [68] in human prostate cancer cells, where carvacrol-induced apoptosis was accompanied by a significant amount of growth arrest in the G0/G1 phase of the cell cycle. This was associated with the decreased expression of cyclin D1 and Cyclin-Dependent Kinase 4 (CDK4) and increased expression of CDK inhibitor p21. CV could inhibit notch signaling in PC-3 cells via downregulating of Notch-1 and Jagged-1 [69].

The various effects of carvacrol on human choriocarcinoma JAR and JEG3 cells lines were investigated. These results revealed that CV halted proliferation and induced apoptosis with an increase in pro-apoptotic proteins in choriocarcinoma cell lines. CV regulates the MAPK and PI3K pathways to induce cell death in both JAR and JEG3 cells (Table 1) [70].

Basbinar and co-workers reported that carvacrol conferred antiproliferative activity on human colorectal carcinoma cell lines HCT-116 and HT-2. The HCT-116 cell line was more resistant to CV treatment compared with the HT-29 cell line. Modified concentrations of soluble factors in the HCT-116 cell line increased the ability to proliferate and survive [71].

Khan et al. prepared carvacrol nanoemulsion (CANE) by using an ultrasonication technique and further evaluated its anticancer potential against human lung adenocarcinoma A549 and PC-9 cells. CANE exhibited strong cytotoxicity in a dose-dependent manner. MTT assay revealed 52.7% cell viability in CANE (100 µg/mL)-treated cells as compared with the untreated A549 cells. The apoptotic potential of a carvacrol nanoemulsion against PC-9 cells was also observed. CANE exhibited dose-dependent cytotoxicity with 62.1 and 52.2% cell viability at 125 and 150 μg/mL, respectively. Furthermore, CANE displayed no cytotoxicity up to 100 µg/mL against healthy bronchial epithelium cells (BEAS-2B). It was found that CANE induced apoptosis in A549 cells by the induction of ROS and exhibited potent antitumor activity in vivo, using an athymic nude mice model [72]. 

The synergistic action of carvacrol and thymol was observed in acute myeloid leukemia (AML) cell lines (KG1, HL60 and K562). These results demonstrated that the combination of carvacrol and thymol induced tumor cell death with low toxicity on healthy cells (PBMC) by activating apoptotic, oxidative and reticular stress pathways [73]. 

**Figure 16:**
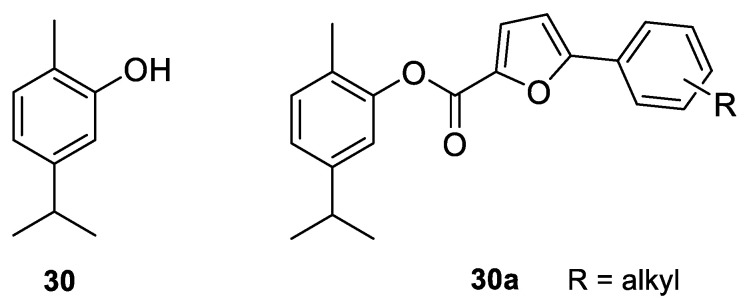
Representative carvacrol derivatives with anticancer activities against Bel-7402 and KB cell lines.

In recent years, full attention has been paid to carvacrol derivatives and their anticancer properties. Novel carvacrol derivatives containing 5-phenyl-2-furan moiety **30a** (Figure 16) were synthesized by Cui and co-workers [74]. All compounds were evaluated for their antitumor activities against Bel-7402 and KB cell lines. These results promise anticancer activity of this carvacrol-based derivative against the aforementioned cell lines. The substituents at the *ortho*- and *para*-positions significantly increased its anticancer activity compared with those at the *meta*-position.

**Figure 17:**
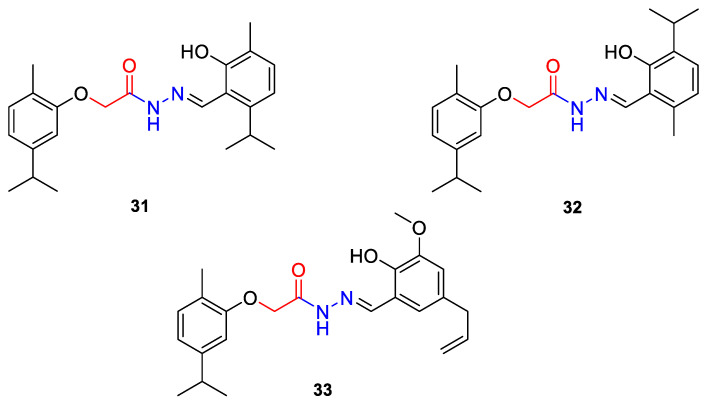
Carvacrol hydrazone derivatives with anticancer potency against pancreatic and colon cancers.

A acetohydrazone analogues of carvacrol **31**–**33** (Figure 17) were synthesized and screened for their in vitro antioxidant activities with DPPH assay and anticancer activities by using sulforhodamine B (SRB) assay against in vitro models of pancreatic cancer (MIAPaCa-2 cell line) and colon cancer (HCT-15 cell line). The tested compounds displayed exquisite antioxidant and anticancer effects. Carvacrylacetohydrazone of eugenol (**33**) possesses excellent cytotoxic potency (GI_50_ = 10 μg/mL) compared with adriamycin (ADR) (GI_50_ = 10 μg/mL) against the HCT-15 and MIAPaCa-2 cell lines (Table 1) [75].

**Figure 18:**
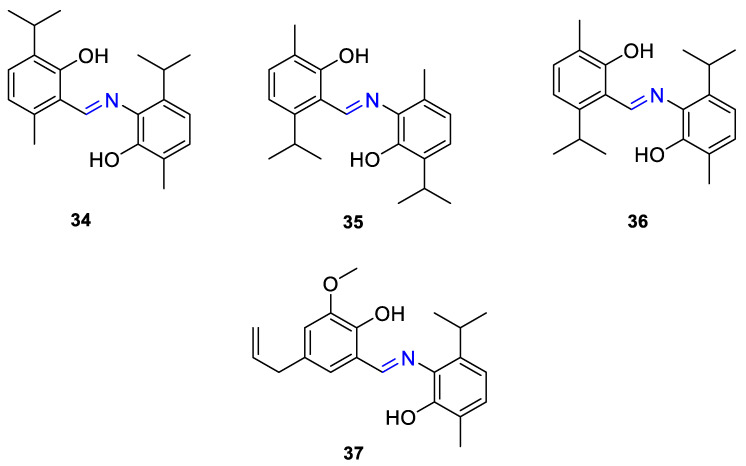
Phenolic monoterpenoids based azomethine scaffolds.

The same group presented the efficient synthesis of carvone-based dihydroxy derivatives of natural phenolic monoterpenoids with azomethine functionality (**34**–**37**) (Figure 18) and evaluated their antioxidant (DPPH assay) and anticancer (SRB assay) potential in pancreatic cancer (MIAPaCa-2) and colon cancer (HCT-15) cell lines. In the antioxidant test, all of the compounds showed strong antioxidant potency. The best results were obtained for (*E*)-4-(2-hydroxy-6-isopropyl-3-methylbenzylideneamino)-2-isopropyl-5-methylphenol **34** with an EC_50_ value significantly lower (0.1171 ± 0.542 μg/mL) than STD (0.1203 ± 0.213 μg/mL). The anticancer test indicated the derivatives possess anticancer efficacy. Specifically, the compound **35** displayed excellent cytotoxic potency (GI_50_ = 10.77 μg/mL) compared with doxorubicin (10 μg/mL) against MIAPaCa-2 cells (Table 1) [76].

Taken together, these data indicate that carvacrol possesses great potential in acting as a chemopreventive agent in cancer research and also in antitumor therapy. In addition, the studies show that carvacrol derivatives retain their strong anticancer activity, e.g., acetohydrazone and imine derivatives with the cytotoxic potency comparable with approved cancer drugs currently in use.

## 7. Menthol and Its Derivatives

**Figure 19:**
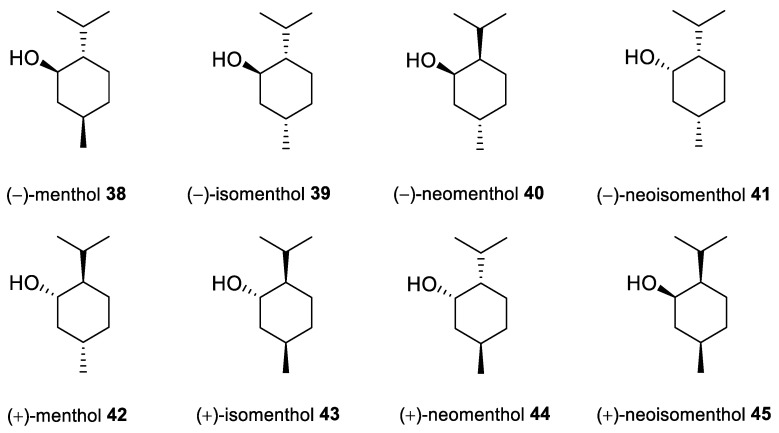
Isomers of menthol.

Menthol [*p*-menthan-3-ol; 5-methyl-2-(1-methylethyl)cyclohexanol; 2-isopropyl-5-methylcyclohexanol], also known as mint camphor, is cyclic monoterpene alcohol that possesses well-known cooling properties and the residual minty smell of the oil remnants from which it is derived [77]. Natural menthol is isolated exclusively from *Mentha canadensis* L. (corn mint) and *Mentha* × *piperita* L. (peppermint; a sterile hybrid of spearmint (*Mentha* × *spicata*) and water mint (*Mentha* × *aquatica*) from the Lamiaceae family), but can also be synthesized on an industrial scale [78]. Menthol exhibits three stereogenic centers, as such, exists in eight stereoisomeric forms (Figure 19). (−)-Menthol, with a 1*R*,3*R*,4*S* configuration from the natural source and synthesized menthol with the same structure is the most preferred isomer, mainly due to its greater cooling properties compared with the other menthol isomers. This monocyclic monoterpene alcohol exhibits antimicrobial, analgesic, antipruritic, antitussive, antiviral and anti-inflammatory properties along with its fumigant and insecticidal activities; therefore, it is widely used in the production of cosmetics, pharmaceuticals and food flavors [78,79].

Menthol exhibits also anticancer properties. Wang et al. [80] established that menthol inhibits the proliferation and motility of prostate cancer cells. The authors concluded that DU145 cells express transient receptor potential melastatin member 8 (TRPM8) and that menthol hinders the proliferation and motility of DU145 cells. Their findings support that the activation of the TRPM8 channel might represent a potential target for the treatment of patients in the late androgen-independent stage with high-level expression of said receptor. Moreover, menthol should also be considered in the treatment of prostate cancer. An independent scientific group [81] studied the role of menthol in the blockade of TRPM8 activity and observed that it reduced the invasion potential of oral squamous carcinoma cell lines. Furthermore, the TRPM8 channel is expressed in human melanoma G-361 cells and channel activation induced by menthol, which should be regarded as a naturally occurring ligand for TRPM8, caused sustainable, incremental increases in both intracellular Ca^2+^ concentration ([Ca^2+^]_i_) and also current amplitude in melanoma cells [82]. The most remarkable finding was that exposure to menthol significantly hindered the survival of melanoma cells. Thus, these results provide a novel profile for the TRPM8 channel that in which Ca^2+^ permeability might be involved in tumor progression in melanoma. The EC_50_ value of menthol on [Ca^2+^]_i_ in human melanoma cells was 286 μM (Table 1). Finally, Kim et al. [79] found that menthol also induces G2/M arrest by downregulating and inhibiting downstream signaling of polo-like kinase 1 (PLK1) in PC-3 prostate cancer cells.

**Figure 20:**
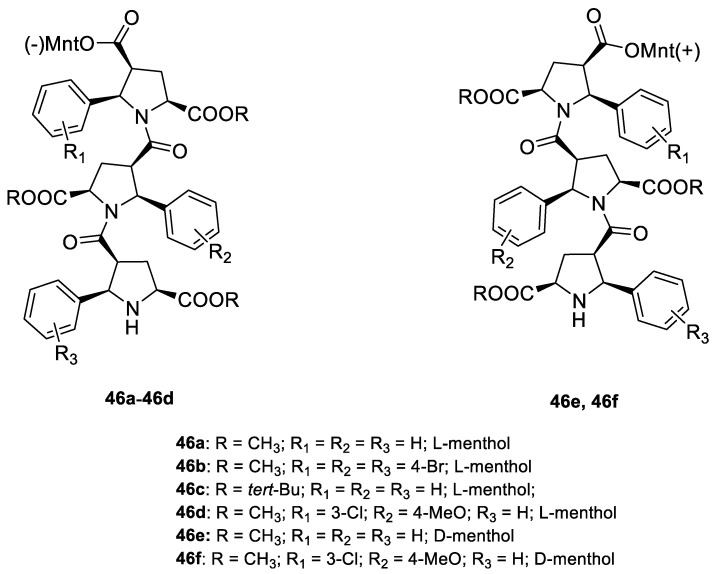
Homochiral 3-PCA trimers with monoterpene menthol derivatives.

Kudryavtsev et al. [83] discovered the anticancer cellular activity of new β-peptides with chiral, active menthol moiety towards hormone-refractory prostate cancer (HRPC) cells. They synthesized a novel class of poly-β-prolines by developing a new method for obtaining chiral β-peptide molecular frameworks based on 1,3-dipolar cycloaddition of azomethine ylides. Sets of pyrrolidine-3-carboxylic acid (3-PCA)-oligomers with up to six pyrrolidine units were produced **46a**–**f** (Figure 20) and their structural and biological properties were comprehensively studied. The effects of 3-PCA oligomers on the proliferation of the HRPC cell line PC-3 were evaluated. The anticancer activity of novel β-peptides against HRPC cells was annotated at low micromolar concentrations; moreover, a dependence on the absolute configuration of the stereogenic centers and the chain length of the β-proline oligomers was observed. Trimer **46e** with the D-menthol moiety was identified as the most active (GI_50_ = 4.4 μM, Table 1).

**Figure 21:**
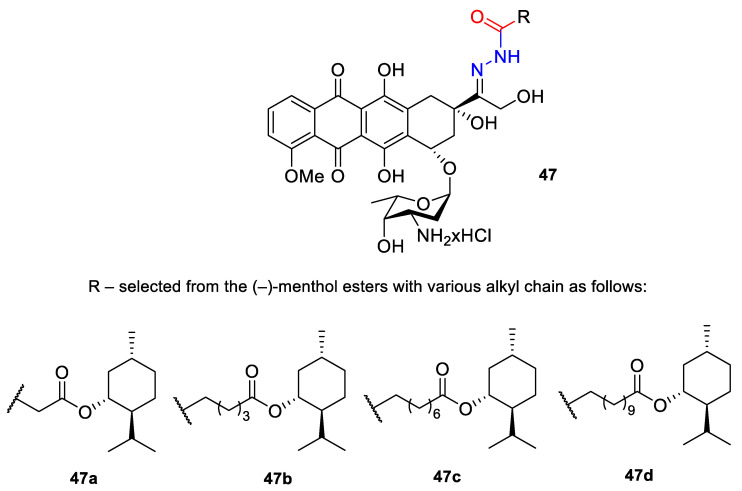
Structures of acyl hydrazones **47** of doxorubicin hydrochloride with (−)-menthol moiety.

A series of *N*-acylhydrazones attached to the C13 of doxorubicin (**47**) (Figure 21) were tested in vitro for eligibility as anticancer agents [84]. The acyl residues were derived from (−)-menthyl esters and incorporated via differentially sized alkyl spacers. These derivatives were assayed for cytotoxicity in cell lines from human cancers that are typically treated with doxorubicin, specifically leukemia, melanoma, breast carcinoma and cervical carcinoma (Table 1). The tested *N*-acylhydrazones of doxorubicin with an added (−)-menthol moiety might complement or even surpass the properties of the parent drug in terms of cytotoxic efficacy, cell specificity, breach of multidrug resistance, mechanism of apoptosis and a combination of molecular targets. The (menthoxycarbonyl)undecanoyl hydrazone compound **47d** was twice as active as doxorubicin against multidrug-resistant KB-V1/Vbl cervical carcinoma and 518A2 melanoma cells. Compound **47d** was also more effective in KB-V1 cervical carcinoma and MCF-7 breast cancer cell lines.

**Figure 22:**
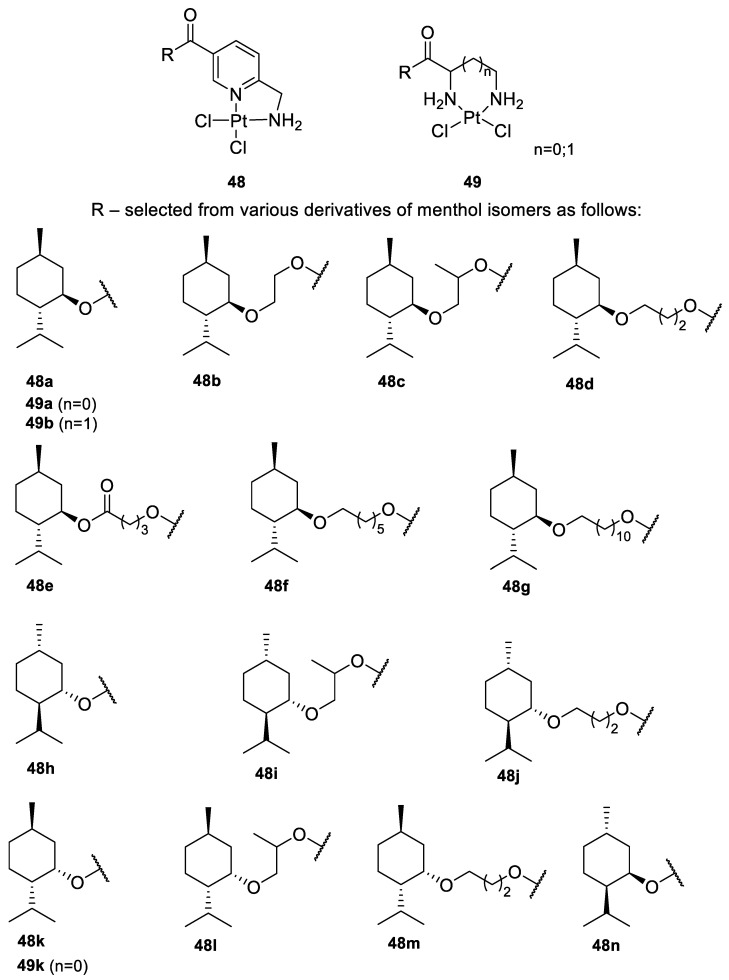
Structures of terpenyl[6-(aminomethyl)nicotinate]dichloridoplatinum(II) **48** and terpenyldichloridoplatinum(II) **49** complexes containing various menthol isomers.

Bernhardt et al. [85] also tested menthol derivatives for antiproliferative activity in human 518A2 melanoma and HL-60 leukemia cells. They synthesized dichloridoplatinum(II) complexes **48** and **49** with several terpenes, including four isomers of menthol moieties, namely (−)-menthol (**48a**–**g**), (+)-menthol (**48h**–**j**), (+)-neomenthol (**48k**–**m**) and (−)-neomenthol (**48n**) and their derivatives (Figure 22). In melanoma cells, the propane-1,2-diyl spaced with a terpene moiety of (−)-menthol derivative **48b** and (+)-neomenthol derivative **48l** displayed growth inhibition at an IC_50_ < 4 µM, which is 10-fold lower than seen with cisplatin (Table 1). Furthermore, presented terpene complexes (**48**, **49**) were tested for antiproliferative activity in human HT-29 colon cells. Greater and more persistent growth inhibition in HT-29 colon cancer cells upon long-term exposure is induced by the (−)-menthyl ester complexes with 2,3-diaminopropanoate (**49a**) and 2,4-diaminobutanoate (**49b**) ligands compared with the 6-(aminomethyl)nicotinate analogue **48a**. The (−)-menthoxyisopropyl ester (**48c**) was the most efficacious against all of the tumor cell lines tested.

The discussed menthol derivatives have very complex structures, including oligomers [83] and complexes [85]. All of the discussed compounds contain functional groups that are commonly used as biologically active molecules, such as ether, ester, imino groups or *N*-acylhydrazones scaffolds [13]. It would be interesting to test antitumor activity in other menthol derivatives that also contain the discussed functional groups but perhaps smaller with less steric hindrance. In this context, interestingly, quaternary ammonium salts and ionic liquids already described as compounds with very strong antimicrobial properties that do not cause hemolysis [86,87].

**Table 1:** Anticancer therapeutic agents of selected monocyclic monoterpene derivatives: carvone, carvacrol, perillyl alcohol, perillaldehyde, limonene and menthol.

No.	Base Unit: Type of the Monocyclic Monoterpenes	Name of Compound Structure	Cancer type Type of Human Tumor Cell Lines	Anticancer Activity Indexes IC_50_; EC_50_; GI; Concentration and Effect on Apoptotic Cell % Cell Viability %	Reference
**1/2**	carvone	(*R*)-(−)-carvone/(*S*)-(+)-carvone 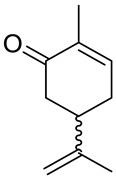	cervical carcinoma (HeLa)	CC_50_ = 74.5 ± 13.1 μg/mL	[20]
**1/2**	carvone	(*R*)-(−)-carvone/(*S*)-(+)-carvone	Vero (healthy green monkey kidney cells)	CC_50_ > 200 μg/mL	[20]
**1/2**	carvone	(*R*)-(−)-carvone/(*S*)-(+)-carvone	murine mastocytoma (P815)	IC_50_ = 0.16 μM	[21]
**1/2**	carvone	(*R*)-(−)-carvone/(*S*)-(+)-carvone	acute lymphoblastic leukemia (CEM)	IC_50_ = 0.11 μM	[21]
**1/2**	carvone	(*R*)-(−)-carvone/(*S*)-(+)-carvone	myelogenous leukemia (K-562)	IC_50_ = 0.17 μM	[21]
**1/2**	carvone	(*R*)-(−)-carvone/(*S*)-(+)-carvone	breast (MCF-7)	IC_50_ = 0.63 μM	[21]
**1/2**	carvone	(*R*)-(−)-carvone/(*S*)-(+)-carvone	breast adenocarcinoma resistant to gemcitabine (MCF-7/gem)	IC_50_ = 0.91 μM	[21]
**1/2**	carvone	(*R*)-(−)-carvone/(*S*)-(+)-carvone	myeloma (KMS-5)	IC_50_ = 20 μM	[27]
**1**	(−)-carvone	(*R*)-(−)-carvone 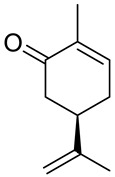	colon (HT29)	IC_50_ = 325 μg/mL (MTT) IC_50_ = 169.5 μg/mL (NR)	[23]
**1**	(−)-carvone	(*R*)-(−)-carvone	CCD 841 CoTr (human healthy colon epithelial cells)	IC_50_ = 475 μg/mL (MTT) IC_50_ = 141.3 μg/mL (NR)	[23]
**1**	(−)-carvone	(*R*)-(−)-carvone	ovarian (OVCAR-8)	GI = 2.28 ± 1.38% at 25 μg/mL	[24]
**1**	(−)-carvone	(*R*)-(−)-carvone	colon (HCT-116)	GI = 11.94 ± 2.54% at 25 μg/mL	[24]
**1**	(−)-carvone	(*R*)-(−)-carvone	brain (SF-295)	GI = 12.28 ± 1.13 at 25 μg/mL	[24]
**2**	(+)-carvone	(*S*)-(+)-carvone 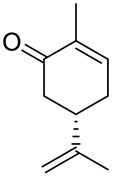	colon (HT29)	IC_50_ nd (MTT) IC_50_ = 106.3 μg/mL (NR)	[23]
**2**	(+)-carvone	(*S*)-(+)-carvone	CCD 841 CoTr (human healthy colon epithelial cells)	IC_50_ = 310 μg/mL (MTT) IC_50_ = 111.2 μg/mL (NR)	[23]
**2**	(+)-carvone	(*S*)-(+)-carvone	ovarian (OVCAR-8)	GI = 48.07 ± 1.20% at 25 μg/mL	[24]
**2**	(+)-carvone	(*S*)-(+)-carvone	colon (HCT-116)	GI = 46.15 ± 2.46% at 25 μg/mL	[24]
**2**	(+)-carvone	(*S*)-(+)-carvone	brain (SF-295)	GI = 34.39 ± 3.47% at 25 μg/mL	[24]
**3a**	(−)-carvone	benzoic acid 2-(4-methyl-5-oxocyclohex-3- enyl)allyl ester 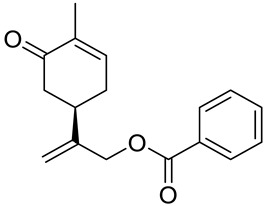	prostate (LNCaP)	GI_50_ > 100 μM	[29]
**3b**	(−)-carvone	4-methylbenzoic acid 2-(4-methyl-5-oxocyclo- hex-3-enyl)allyl ester 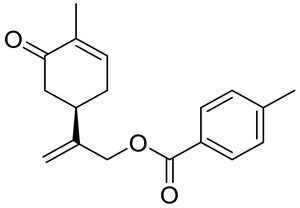	prostate (LNCaP)	GI_50_ = 57 μM	[29]
**3c**	(−)-carvone	4-fluorobenzoic acid 2-(4-methyl-5-oxocyclo- hex-3-enyl)allyl ester 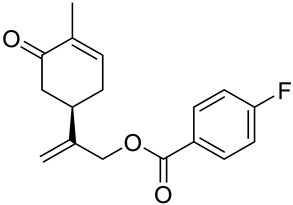	prostate (LNCaP)	GI_50_ >100 μM	[29]
**3d**	(−)-carvone	4-chlorobenzoic acid 2-(4-methyl-5-oxocyclo- hex-3-enyl)allyl ester 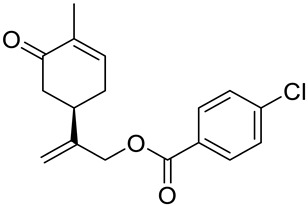	prostate (LNCaP)	GI_50_ = 92 μM	[29]
**3e**	(−)-carvone	4-bromobenzoic acid 2-(4-methyl-5-oxocyclo- hex-3-enyl)allyl ester 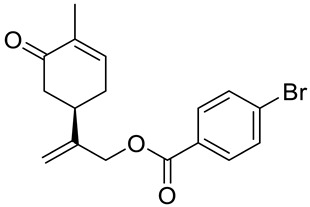	prostate (LNCaP)	GI_50_ = 80 μM	[29]
**3f**	(−)-carvone	4-methoxybenzoic acid 2-(4-methyl-5-oxocy- clohex-3-enyl)allyl ester 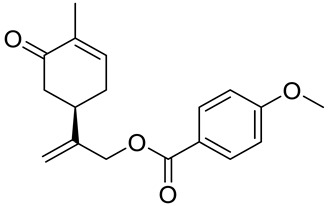	prostate (LNCaP)	GI_50_ = 21 μM	[29]
**3g**	(−)-carvone	4-aminobenzoic acid 2-(4-methyl-5-oxocyclo- hex-3-enyl)allyl ester 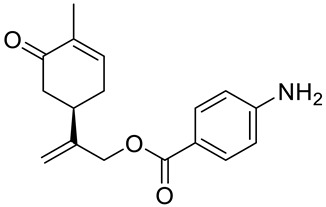	prostate (LNCaP)	GI_50_ = 45 μM	[29]
**4a**	(−)-carvone	2-methyl-5-{[1-(4-methylpiperazin-1-yl)meth- yl]vinyl}cyclohex-2-enone 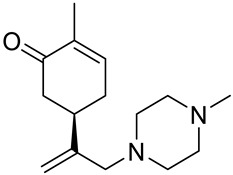	prostate (LNCaP)	GI_50_ > 100 μM	[29]
**4b**	(−)-carvone	5-[1-(4-ethylpiperazin-1-yl)methyl]vinyl-2- methylcyclohex-2-enone 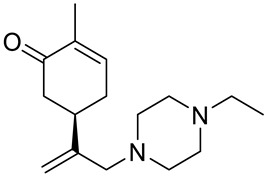	prostate (LNCaP)	GI_50_ > 100 μM	[29]
**4c**	(−)-carvone	5-[1-(4-isopropylpiperazin-1-yl)methyl]vinyl-2-methylcyclohex-2-enone 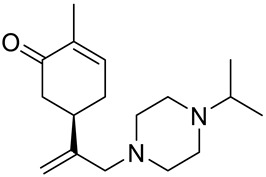	prostate (LNCaP)	GI_50_ > 100 μM	[29]
**4d**	(−)-carvone	5-[1-(4-isobutylpiperazin-1-yl)methyl]vinyl-2- methylcyclohex-2-enone 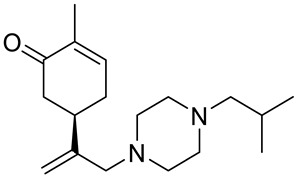	prostate (LNCaP)	GI_50_ > 100 μM	[29]
**4e**	(−)-carvone	5-[1-(4-benzylpiperazin-1-yl)methyl]vinyl-2- methylcyclohex-2-enone 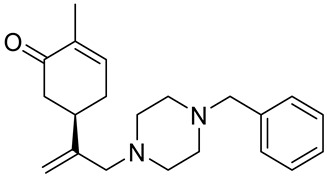	prostate (LNCaP)	GI_50_ > 100 μM	[29]
**4f**	(−)-carvone	5-{1-[4-(4-methoxyphenyl)piperazin-1-yl]- methyl}vinyl-2-methylcyclohex-2-enone 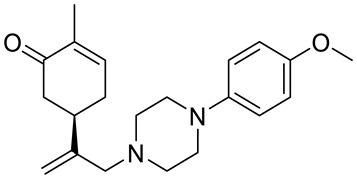	prostate (LNCaP)	GI_50_ = 45 μM	[29]
**4g**	(−)-carvone	5-{1-[4-(2-methoxyphenyl)piperazin-1-yl]- methyl}vinyl-2-methylcyclohex-2-enon 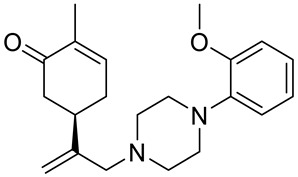	prostate (LNCaP)	GI_50_ = 37 μM	[29]
**4h**	(−)-carvone	5-{1-[4-(2-chlorophenyl)piperazin-1-yl]meth- yl}vinyl-2-methylcyclohex-2-enone 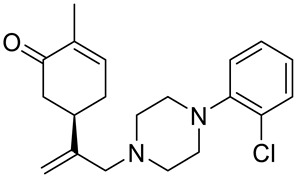	prostate (LNCaP)	GI_50_ = 19 μM	[29]
**5a**	(−)-carvone	2-methyl-5-[1-(pyrrolidin-1-ylmethyl)vinyl]- cyclohex-2-enone 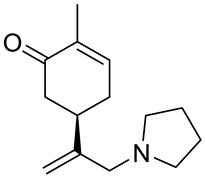	prostate (LNCaP)	GI_50_ > 100 μM	[29]
**5b**	(−)-carvone	2-methyl-5-[1-(piperidin-1-ylmethyl)vinyl]- cyclohex-2-enone 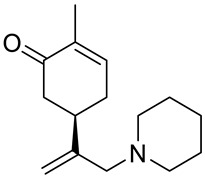	prostate (LNCaP)	GI_50_ > 100 μM	[29]
**5c**	(−)-carvone	5-(1-cyclohexylaminomethyl)vinyl-2-methyl- cyclohex-2-enone 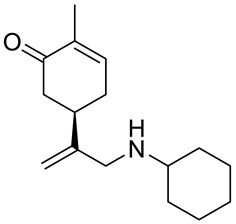	prostate (LNCaP)	GI_50_ > 100 μM	[29]
**5d**	(−)-carvone	2-methyl-5-{1-[(2-thiophen-2-ylethylamino)- methyl]vinyl}cyclohex-2-enone 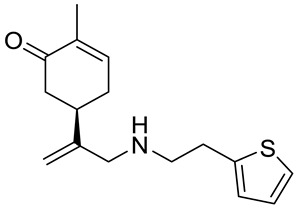	prostate (LNCaP)	GI_50_ = 24 μM	[29]
**5e**	(−)-carvone	5-(1-dimethylaminomethyl)vinyl-2-methylcy- clohex-2-enone 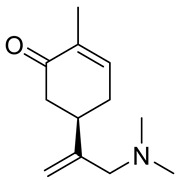	prostate (LNCaP)	GI_50_ = 75 μM	[29]
**5f**	(−)-carvone	5-[1-(adamantan-1-ylamino)methyl]vinyl-2- methylcyclohex-2-enone 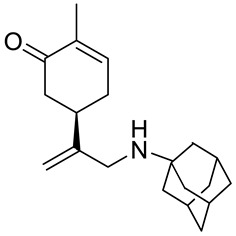	prostate (LNCaP)	GI_50_ = 83 μM	[29]
**7**	(+)-carvone	hydroisobenzofuran derivative of (+)-carvone (ester) 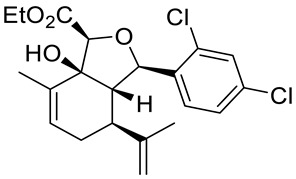	epithelial carcinoma (KB-3)	IC_50_ = 3 μM	[30]
**8**	(+)-carvone	hydroisobenzofuran derivative of (+)-carvone (diene) 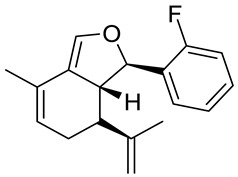	epithelial carcinoma (KB-3)	IC_50_ = 1 μM	[30]
**8**	(+)-carvone	hydroisobenzofuran derivative of (+)-carvone (diene)	leukemia (RPMI-8226)	GI_50_ = 0.148 μM LC_50_ = 9.36 μM	[30]
**8**	(+)-carvone	hydroisobenzofuran derivative of (+)-carvone (diene)	lung (HOP-92	GI_50_ = 0.552 μM LC_50_ = 26.8 μM	[30]
**9**	(+)-carvone	hydroisobenzofuran derivative of (+)-carvone (enone) 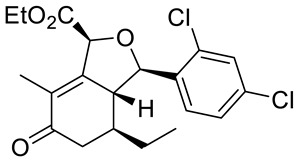	epithelial carcinoma (KB-3)	IC_50_ = 3 μM	[30]
**10**	(−)-carvone	(−)-8-hydroxycarvotanacetone 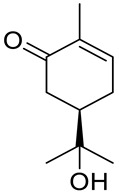	colon (HCT-116)	GI = 75.2%	[24]
**10**	(−)-carvone	(−)-8-hydroxycarvotanacetone	ovarian (OVCAR-8)	GI = 94.01%	[24]
**10**	(−)-carvone	(−)-8-hydroxycarvotanacetone	brain (SF-295)	GI = 61.59%	[24]
**11**	(+)-carvone	(+)-8-hydroxycarvotanacetone 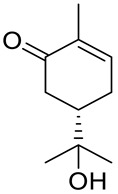	colon (HCT-116)	GI = 4.76%	[24]
**11**	(+)-carvone	(+)-8-hydroxycarvotanacetone	ovarian (OVCAR-8)	GI = 3.12%	[24]
**11**	(+)-carvone	(+)-8-hydroxycarvotanacetone	brain (SF-295)	GI = 16.36%	[24]
**12**	(−)-carvone	(−)-carvone epoxide 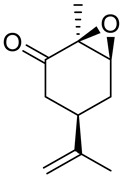	colon (HCT-116)	GI = 29.24%	[24]
**12**	(−)-carvone	(−)-carvone epoxide	ovarian (OVCAR-8)	GI = 8.21%	[24]
**12**	(−)-carvone	(−)-carvone epoxide	brain (SF-295)	GI = 10.93%	[24]
**13**	(−)-carvone	(−)-8-acetoxycarvotanacetone 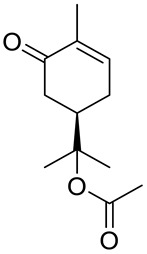	colon (HCT-116)	GI = 10.36%	[24]
**13**	(−)-carvone	(−)-8-acetoxycarvotanacetone	ovarian (OVCAR-8)	GI = 1.62%	[24]
**13**	(−)-carvone	(−)-8-acetoxycarvotanacetone	brain (SF-295)	GI = 30.47%	[24]
**14**	(−)-carvone	(*R*,*E*)-2-(2-methyl-5-(prop-1-en-2-yl)cyclohex-2-en-1-one)thiosemicarbazone 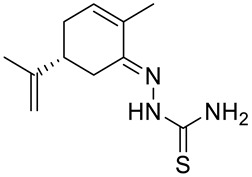	larynx epidermoid carcinoma (Hep2)	the number of dead and apoptotic cells increased to about 20% at concentration 40 μM	[33]
**15**	(−)-carvone	complex Pd_2_L_2_Cl_4_; L = (*R*)-(−)-carvone 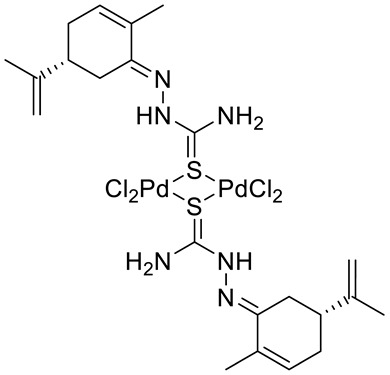	larynx epidermoid carcinoma (Hep2)	IC_50_ = 30 μM	[33]
**17**	(+)-limonene	(*R*)-(+)-limonene 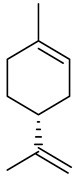	bladder	concentration 36 µM give 34.71% apoptotic cell percentage	[37,88]
**17**	(+)-limonene	(*R*)-(+)-limonene	lung (A549, H1299)	cells showed increase in the expression of autophagy- related genes lc3b, beclin1, atg3, atg5, atg7, atg12 and atg14 with enhanced protein expressions of the autophagy-related proteins LC3-II and Atg5	[37,89]
**18**	(+)-limonene	(+)-limonene 1,2-epoxide 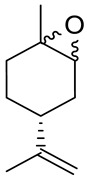	colon (HCT-116)	GI = 73.13%	[24]
**18**	(+)-limonene	(+)-limonene 1,2-epoxide	ovarian (OVCAR-8)	GI = 93.1%	[24]
**18**	(+)-limonene	(+)-limonene 1,2-epoxide	brain (SF-295)	GI = 58.48%	[24]
**18**	(+)-limonene	solid lipid nanoparticles (SLNs) formulation with (+)-limonene 1,2-epoxide 18 and glycerol monostearate (LIM-SLNs)	human skin (HaCaT)	cell viability 76.27 ± 1.63%	[39]
**19**	(+)-limonene	(+)-(1*S*,2*S*,4*R*)-limonene-1,2-diol 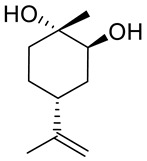	lung (A549)	IC_50_ = 1.53 – 0.04 mg/mL (48 h)	[40]
**19**	(+)-limonene	(+)-(1*S*,2*S*,4*R*)-limonene-1,2-diol	lung (H1264)	IC_50_ = 1.73 ± 0.04 mg/mL (48 h)	[40]
**19**	(+)-limonene	(+)-(1*S*,2*S*,4*R*)-limonene-1,2-diol	lung (H1299)	IC_50_ = 1.39 ± 0.06 mg/mL (48 h)	[40]
**19**	(+)-limonene	(+)-(1*S*,2*S*,4*R*)-limonene-1,2-diol	lung (Calu-6)	IC_50_ = 0.62 ± 0.02 mg/mL (48 h)	[40]
**20**	(−)-perillyl alcohol	POH/β-CD	sarcoma (S180)	GI = 60%	[46]
**22**	(−)-perillyl alcohol	[(4*S*)-4-prop-1-en-2-ylcyclohexen-1-yl]methyl *N*-(3-methyl-4-oxoimidazo[5,1-d][1,2,3,5]tetrazine-8-carbonyl)carbamate (NEO212) 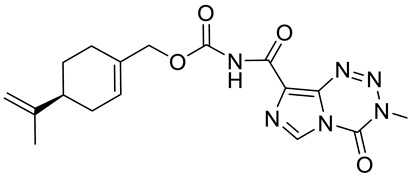	lymphoma (HUT-78)	IC_50_ = 8 μM (24 h) IC_50_ ≤ 3 μM (48 h)	[47]
**22**	(−)-perillyl alcohol	[(4*S*)-4-prop-1-en-2-ylcyclohexen-1-yl]methyl *N*-(3-methyl-4-oxoimidazo[5,1-d][1,2,3,5]tetrazine-8-carbonyl)carbamate (NEO212)	lymphoma (HUT-102)	IC_50_ = 9 μM (72 h) IC_50_ = 3 μM (96 h)	[47]
**22**	(−)-perillyl alcohol	[(4*S*)-4-prop-1-en-2-ylcyclohexen-1-yl]methyl *N*-(3-methyl-4-oxoimidazo[5,1-d][1,2,3,5]tetrazine-8-carbonyl)carbamate (NEO212)	lymphoma (MyLa)	IC_50_ = 130 μM (72 h) IC_50_ = 85 μM (96 h)	[47]
**23**	(−)-perillyl alcohol	perillyl alcohol/temozolomide/linoleic acid conjugate (NEO412) 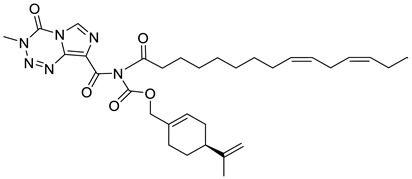	melanoma (A2058, M24)	IC_50_ = 5 μM	[48]
**23**	(−)-perillyl alcohol	perillyl alcohol/temozolomide/linoleic acid conjugate (NEO412)	melanoma (A375, M249)	IC_50_ = 25–35 μM	[48]
**24**	(−)-perillyl alcohol	4-(prop-1-en-2-yl)-*N*-(3-(trifluoromethyl)phenyl)cyclohex-1-ene-1-carboxamide 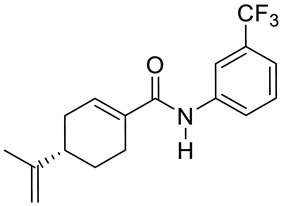	glioblastoma (U251)	IC_50_ = 9.41 ± 0.38 μM	[49]
**24**	(−)-perillyl alcohol	4-(prop-1-en-2-yl)-*N*-(3-(trifluoromethyl)phenyl)cyclohex-1-ene- 1-carboxamide	hepatocellular carcinoma (HepG2)	IC_50_ = 18.07 ± 0.10 μM	[49]
**25**	(−)-perillyl alcohol	*N*-(4-(4-amino-2-methylphenethyl)-3-methylphenyl)-4-(prop-1-en-2-yl)cyclohex-1-ene-1-carboxamide 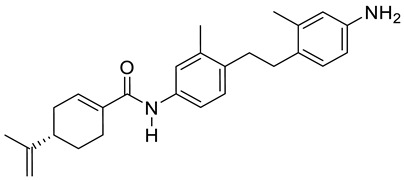	glioblastoma (U251)	IC_50_ = 3.10 ± 0.12 μM	[49]
**25**	(−)-perillyl alcohol	*N*-(4-(4-amino-2-methylphenethyl)-3-methylphenyl)-4-(prop-1- en-2-yl)cyclohex-1-ene-1-carboxamide	hepatocellular carcinoma (HepG2)	IC_50_ = 1.49 ± 0.43 μM	[49]
**26a**	(−)-perillyl alcohol	4-{[(4*S*)-4-isopropenylcyclohex-1-en-1-yl]methyl}benzene-1,2-diol 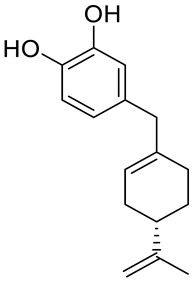	breast (MCF-7)	IC_50_ = 25.9 ± 0.1 μM	[50]
**26a**	(−)-perillyl alcohol	4-{[(4*S*)-4-isopropenylcyclohex-1-en-1-yl]methyl}benzene-1,2-diol	prostate (PC-3)	IC_50_ = 12.2 ± 0.7 μM	[50]
**26a**	(−)-perillyl alcohol	4-{[(4*S*)-4-isopropenylcyclohex-1-en-1-yl]methyl}benzene-1,2-diol	colon (HT-29)	IC_50_ = 45.1 ± 0.2 μM	[50]
**26b**	(−)-perillyl alcohol	4-{[(4*S*)-4-isopropenylcyclohex-1-en-1-yl]methyl}benzene-1,3-diol 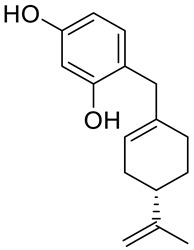	breast (MCF-7)	IC_50_ = 53.7 ± 0.4 μM	[50]
**26b**	(−)-perillyl alcohol	4-{[(4*S*)-4-isopropenylcyclohex-1-en-1-yl]methyl}benzene-1,3-diol	prostate (PC-3)	IC_50_ = 54.5 ± 0.5 μM	[50]
**26c**	(−)-perillyl alcohol	3-{[(4*S*)-4-isopropenylcyclohex-1-en-1-yl]methoxy}phenol 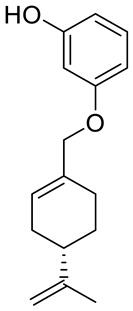	breast (MCF-7)	IC_50_ = 44.3 ± 0.7 μM	[50]
**26c**	(−)-perillyl alcohol	3-{[(4*S*)-4-isopropenylcyclohex-1-en-1-yl]methoxy}phenol	prostate (PC-3)	IC_50_ = 79.0 ± 0.2 μM	[50]
**27a**	(−)-perillaldehyde	(*S*)-*N*-((4-(prop-1-en-2-yl)cyclohex-1-enyl)methyl)amantadine 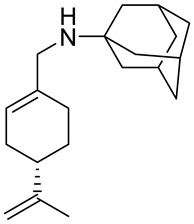	lung (A549)	IC_50_ = 53.80 μM	[54]
**27a**	(−)-perillaldehyde	(*S*)-*N*-((4-(prop-1-en-2-yl)cyclohex-1-enyl)methyl)amantadine	melanoma (A375-S2)	IC_50_ = 53.80 μM	[54]
**27a**	(−)-perillaldehyde	(*S*)-*N*-((4-(prop-1-en-2-yl)cyclohex-1-enyl)methyl)amantadine	fibrosarcoma (HT1080)	IC_50_ = 56.17 μM	[54]
**27b**	(−)-perillaldehyde	(*S*)-*N*-((4-(prop-1-en-2-yl)cyclohex-1-enyl)methyl)cyclohexanamine 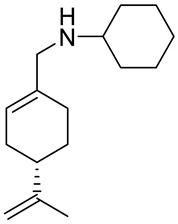	lung (A549)	IC_50_ = 69.50 μM	[54]
**27b**	(−)-perillaldehyde	(*S*)-*N*-((4-(prop-1-en-2-yl)cyclohex-1-enyl)methyl)cyclohexanamine	melanoma (A375-S2)	IC_50_ = 72.77 μM	[54]
**27b**	(−)-perillaldehyde	(*S*)-*N*-((4-(prop-1-en-2-yl)cyclohex-1-enyl)methyl)cyclohexanamine	fibrosarcoma (HT1080)	IC_50_ = 69.37 μM	[54]
**28**	(−)-perillaldehyde	(−)-perillaldehyde 1,2-epoxide 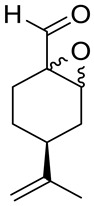	Colon (HCT-116)	GI = 99.46 ± 1.54% IC_50_ = 16.14 ± 1.86 μM	[55]
**28**	(−)-perillaldehyde	(−)-perillaldehyde 1,2-epoxide	ovarian (OVCAR-8)	GI = 99.37 ± 0.30% IC_50_ = 23.61 ± 1.13 μM	[55]
**28**	(−)-perillaldehyde	(−)-perillaldehyde 1,2-epoxide	glioblastoma (SF-295)	GI = 95.66 ± 5.06% IC_50_ = 21.99 ± 2.64 μM	[55]
**28**	(−)-perillaldehyde	(−)-perillaldehyde 1,2-epoxide	leukemia (HL-60)	GI = 99.71 ± 2.43% IC_50_ = 9.70 ± 1.01 μM	[55]
**28**	(−)-perillaldehyde	cSLNs loaded with perillaldehyde 1,2-epoxide	breast (MCF-7)	IC50 = 195.08 μg/mL	[56]
**29**	(−)-perillaldehyde	(−)-perillaldehyde 8,9-epoxide 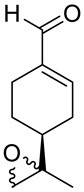	colon (HCT-116)	GI = 98.64 ± 0.74% IC_50_ = 1.03 μM	[24]
**29**	(−)-perillaldehyde	(−)-perillaldehyde 8,9-epoxide	ovarian (OVCAR-8)	GI = 96.32 ± 1.51% IC_50_ = 1.15 μM	[24]
**29**	(−)-perillaldehyde	(−)-perillaldehyde 8,9-epoxide	glioblastoma (SF-295)	GI = 99.89 ± 0.24% IC_50_ = 1.75 μM	[24]
**29**	(−)-perillaldehyde	(−)-perillaldehyde 8,9-epoxide	leukemia (HL-60)	IC_50_ = 0.64 μM	[24]
**30**	carvacrol	carvacrol 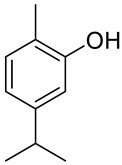	metastatic breast (MDA-MB 231)	IC_50_ = 100 μM IC_50_ = 199 μM	[59] [60]
**30**	carvacrol	carvacrol	glioblastoma (U87)	IC_50_ = 322 μM	[60]
**30**	carvacrol	carvacrol	hepatocellular carcinoma (HepG2)	IC_50_ = 53.09 μg/mL IC_50_ = 0.4 mmol/L IC_50_ = 48.3 mg/L	[63] [64] [65]
**30**	carvacrol	carvacrol	L02 (human healthy epatocyte line)	cell viability is 100% with 0.4 mmol/L	[64]
**30**	carvacrol	carvacrol	HEK293 (healthy human renal cells)	IC_50_ = 90.5 mg/L	[65]
**30**	carvacrol	carvacrol	murine mastocytoma (P815)	IC_50_ = 0.067 μM	[21]
**30**	carvacrol	carvacrol	acute lymphoblastic leukemia (CEM)	IC_50_ = 0.042 μM	[21]
**30**	carvacrol	carvacrol	K-562 (human chronic myelogenous leukemia)	IC_50_ = 0.067 μM	[21]
**30**	carvacrol	carvacrol	breast (MCF-7)	IC_50_ = 0.125 μM	[21]
**30**	carvacrol	carvacrol	breast adenocarcinoma resistant to gemcitabine (MCF-7/gem)	0.067 μM	[21]
**30**	carvacrol	carvacrol	choriocarcinoma ( JAR)	the cell viability decreased (76%) and increased the population of late apoptotic cells (23.8%) at concentration 300 µM	[70]
**30**	carvacrol	carvacrol	choriocarcinoma ( JEG3)	the cell viability decreased (49%) and increased the population of late apoptotic cells (1023%) at concentration 300 µM	[70]
**30**	carvacrol	carvacrol	colon (HCT-116)	IC_50_ = 92 µM (48 h)	[71]
**30**	carvacrol	carvacrol	colon (HT-29)	IC_50_ = 42 µM (48 h)	[71]
**30**	carvacrol	carvacrol nanoemulsion CANE	lung (A549)	52.7% cell viability at concentration 100 µg/mL; 40.5% sub-G1 cell accumulation was observed at 100 µg/mL of CANE treatment	[72]
**30**	carvacrol	carvacrol nanoemulsion CANE	lung (PC-9)	dose-dependent cytotoxicity with 62.1 and 52.2% cell viability at 125 and 150 μg/mL concentrations	[72]
**30**	carvacrol	carvacrol nanoemulsion CANE	BEAS-2B (healthy bronchial epithelium cells)	no cytotoxicity up to 100 µg/ml	[72]
**30**	carvacrol	carvacrol nanoemulsion CANE	tumor in mice	34.2 and 62.1% reduction in tumor weight in the mice treated with 50 and 100 mg/kg CANE	[72]
**30**	carvacrol	carvacrol	leukemia (KG1)	60% cell viability at concentration 300 µg/mL (24 h) and 30% (48 h); the combination of car-vacrol/thymol (300 μM/50 μM): 10% cell viability	[73]
**30**	carvacrol	carvacrol	leukemia (HL60)	80% cell viability at concentration 300 µg/mL (24 h) and 60% (48 h); the combination of car-vacrol/thymol (300 μM/50 μM): 5% cell viability	[73]
**30**	carvacrol	carvacrol	myelogenous leukemia (K562)	80% cell viability at concentration 300 µg/mL (24 h); the combination of carvacrol/thymol (300 μM/50 μM): 30% cell viability	[73]
**30**	carvacrol	carvacrol	(PBMCs) peripheral blood mononuclear cell from healthy donors	65% cell viability at concentration 300 µg/mL (48 h); the combination of carvacrol/thymol (300 μM/50 μM): 55% cell viability	[73]
**31**	carvacrol	(*E*)-*N*-(2-hydroxy-6-isoproyl-3-methylbenzylindine)-2-(5-isopropyl-2-methylphenoxy) acetohydrazide 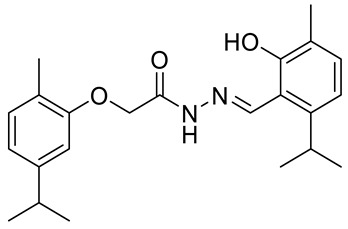	colon (HCT-15)	GI_50_ = 80 µg/mL	[75]
**31**	carvacrol	(*E*)-*N*-(2-hydroxy-6-isoproyl-3-methylbenzylindine)-2-(5-isopropyl-2-methylphenoxy) acetohydrazide	pancreatic (MIPaCa-2)	GI_50_ = 80 µg/mL	[75]
**32**	carvacrol	(*E*)-*N*-(2-hydroxy-3-isoproyl-6-methylbenzylindine)-2-(5-isopropyl-2-methylphenoxy) acetohydrazide 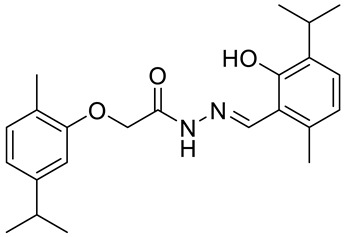	colon (HCT-15)	GI_50_ = 80 µg/mL	[75]
**32**	carvacrol	(*E*)-*N*-(2-hydroxy-3-isoproyl-6-methylbenzylindine)-2-(5-isopropyl-2-methylphenoxy) acetohydrazide	pancreatic (MIPaCa-2)	GI_50_ = 10 µg/mL	[75]
**33**	carvacrol	(*E*)-*N*-(5-allyl-2-hydroxy-3-methoxybenzylindine)-2-(5-isopropyl-2-methylphenoxy) acetohydrazide 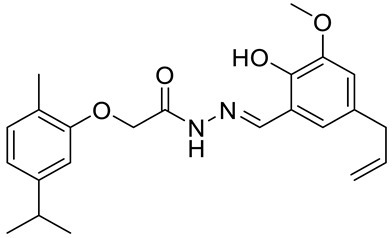	colon (HCT-15)	GI_50_ = 10 µg/mL	[75]
**33**	carvacrol	(*E*)-*N*-(5-allyl-2-hydroxy-3-methoxybenzylindine)-2-(5-isopropyl-2-methylphenoxy) acetohydrazide	pancreatic (MIPaCa-2)	GI_50_ = 10 µg/mL	[75]
**34**	carvacrol	(*E*)-4-(2-hydroxy-6-isopropyl-3-methylbenzylideneamino)-2-isopropyl-5-methylphenol 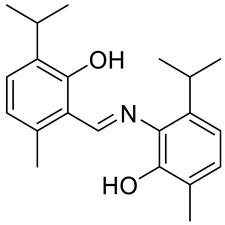	colon (HCT-15)	GI_50_ = 80 µg/mL	[76]
**34**	carvacrol	(*E*)-4-(2-hydroxy-6-isopropyl-3-methylbenzylideneamino)-2-isopropyl-5-methylphenol	pancreatic (MIPaCa-2)	GI_50_ = 80 µg/mL	[76]
**35**	carvacrol	(*E*)-4-(2-hydroxy-3-isopropyl-6-methylbenzylideneamino)-5-isopropyl-2-methylphenol 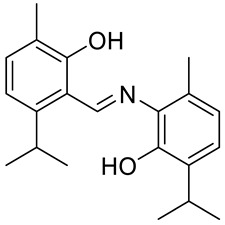	colon (HCT-15)	GI_50_ = 80 µg/mL	[76]
**35**	carvacrol	(*E*)-4-(2-hydroxy-3-isopropyl-6-methylbenzylideneamino)-5-isopropyl-2-methylphenol	pancreatic (MIPaCa-2)	GI_50_ = 10.77 µg/mL	[76]
**36**	carvacrol	(*E*)-4-(2-hydroxy-6-isopropyl-3-methylbenzylideneamino)-5-isopropyl-2-methylphenol 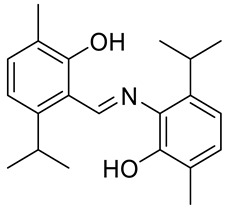	colon (HCT-15)	GI_50_ = 80 µg/mL	[76]
**36**	carvacrol	(*E*)-4-(2-hydroxy-6-isopropyl-3-methylbenzylideneamino)-5-isopropyl-2-methylphenol	pancreatic (MIPaCa-2)	GI_50_ = 16.9 µg/mL	[76]
**37**	carvacrol	(*E*)-4-(5-allyl-2-hydroxy-3-methoxybenzylideneamino)-5-isopropyl-2-methylphenol 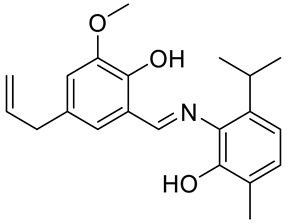	colon (HCT-15)	GI_50_ = 80 µg/mL	[76]
**37**	carvacrol	(*E*)-4-(5-allyl-2-hydroxy-3-methoxybenzylideneamino)-5-isopropyl-2-methylphenol	pancreatic (MIPaCa-2)	GI_50_ = 16.5 µg/ml	[76]
**38**	(−)-menthol	(−)-menthol 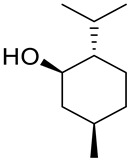	melanoma (G-361)	EC_50_ = 286 μM IC_50_ = 682 μM	[82]
**46a**	(−)-menthol	4-((1*R*,2*S*,5*R*)-2-isopropyl-5-methylcyclohexyl) 2-methyl (2S,4*S*,5*R*)-1-((2*S*,3*R*,5*R*)-5-(methoxycarbonyl)-1-((2*R*,3*S*,5*S*)-5-(methoxycarbonyl)-2-phenylpyrrolidine-3-carbonyl)-2-phenylpyrrolidine-3-carbonyl)-5-phenylpyrrolidine-2,4-dicarboxylate 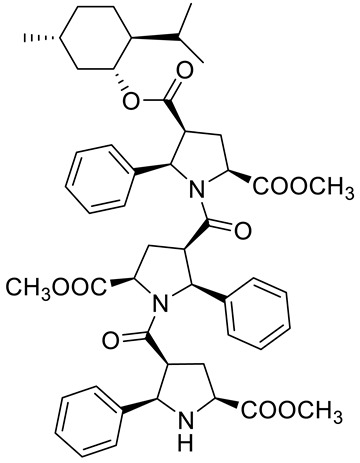	prostate (PC-3)	GI_50_ = 6.4 µM	[83]
**46b**	(−)-menthol	4-((1*R*,2*S*,5*R*)-2-isopropyl-5-methylcyclohexyl) 2-methyl (2*S*,4*S*,5*R*)-5-(4-bromophenyl)-1-((2*S*,3*R*,5*R*)-2-(4-bromophenyl)-1-((2*R*,3*S*,5*S*)-2-(4-bromophenyl)-5-(methoxycarbonyl)-pyrrolidine-3-carbonyl)-5-(methoxycarbonyl)pyrrolidine-3-carbonyl)pyrrolidine-2,4-dicarboxylate 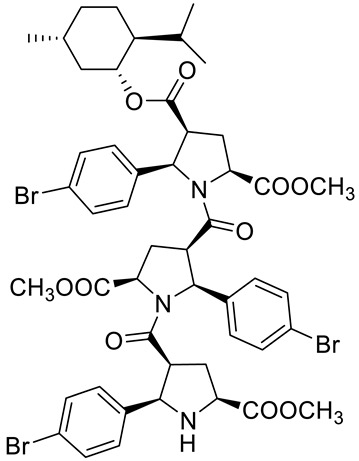	prostate (PC-3)	GI_50_ = 30.0 µM	[83]
**46c**	(−)-menthol	2-(*tert*-butyl) 4-((1*R*,2*S*,5*R*)-2-isopropyl-5-methylcyclohexyl) (2*S*,4*S*,5*R*)-1-((2*S*,3*R*,5*R*)-5-(*tert*-butoxycarbonyl)-1-((2*R*,3*S*,5*S*)-5-(*tert*-butoxycarbonyl)-2-phenylpyrrolidine-3-carbonyl)-2-phenylpyrrolidine-3-carbonyl)-5-phenylpyrrolidine-2,4-dicarboxylate 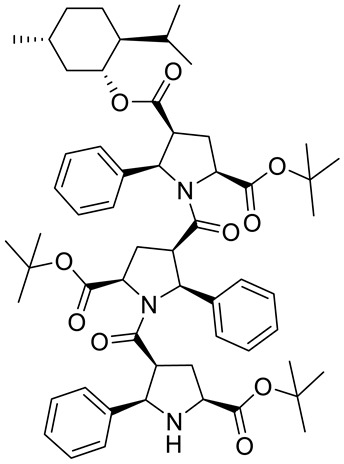	prostate (PC-3)	GI_50_ > 30.0 µM	[83]
**46d**	(−)-menthol	4-((1*R*,2*S*,5*R*)-2-isopropyl-5-methylcyclohexyl) 2-methyl (2*S*,4*S*,5*R*)-5-(3-chlorophenyl)-1-((2*S*,3*R*,5*R*)-5-(methoxycarbonyl)-1-((2*R*,3S,5*S*)-5-(methoxycarbonyl)-2-phenylpyrrolidine-3- carbonyl)-2-(4-methoxyphenyl)pyrrolidine-3-carbonyl)pyrrolidine-2,4-dicarboxylate 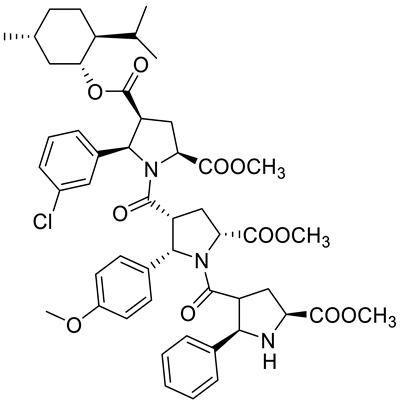	prostate (PC-3)	GI_50_ = 5.3 µM	[83]
**46e**	(+)-menthol	4-((1*S*,2*R*,5*S*)-2-isopropyl-5-methylcyclohexyl) 2-methyl (2*R*,4*R*,5*S*)-1-((2*R*,3*S*,5*S*)-5-(methoxycarbonyl)-1-((2*S*,3*R*,5*R*)-5-(methoxycarbonyl)-2-phenylpyrrolidine-3-carbonyl)-2- phenylpyrrolidine-3-carbonyl)-5-phenylpyrrolidine-2,4-dicarboxylate 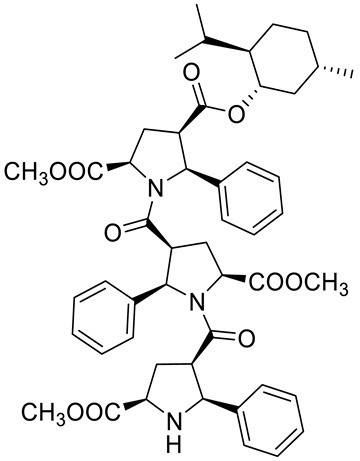	prostate (PC-3)	GI_50_ = 4.4 μM	[83]
**46f**	(+)-menthol	4-((1*S*,2*R*,5*S*)-2-isopropyl-5-methylcyclohexyl) 2-methyl (2*R*,4*R*,5*S*)-5-(3-chlorophenyl)-1-((2R,3S,5S)-5-(methoxycarbonyl)-1-((2*S*,3*R*,5*R*)-5-(methoxycarbonyl)-2-phenylpyrrolidine-3-carbonyl)-2-(4-methoxyphenyl)pyrrolidine-3-carbonyl)pyrrolidine-2,4-dicarboxylate 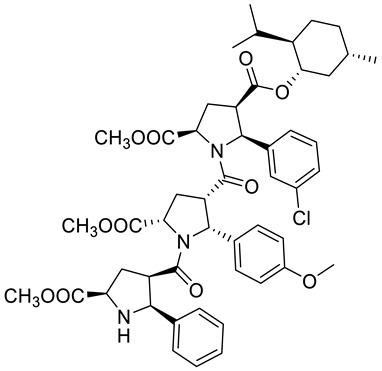	prostate (PC-3)	GI_50_ = 6.0 μM	[83]
**47a**	(−)-menthol	doxorubicin menthoxycarbonylacetylhydrazone 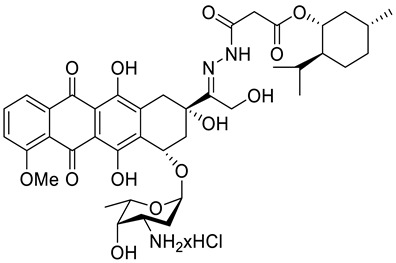	leukemia (HL-60)	IC_50_ = 0.57 ± 0.18 μM (24 h) IC_50_ = 0.26 ± 0.11 μM (48 h) IC_50_ = 0.23 ± 0.10 μM (72 h)	[84]
**47a**	(−)-menthol	doxorubicin menthoxycarbonylacetylhydrazone	melanoma (518A2)	IC_50_ = 0.71 ± 0.52 μM (24 h) IC_50_ = 0.54 ± 0.02 μM (48 h) IC_50_ = 0.14 ± 0.01 μM (72 h)	[84]
**47a**	(−)-menthol	doxorubicin menthoxycarbonylacetylhydrazone	breast (MCF-7/Topo)	IC_50_ = 8.4 ± 2.6 μM (24 h) IC_50_ = 3.8 ± 1.0 μM (48 h) IC_50_ = 2.7 ± 1.0 μM (72 h)	[84]
**47a**	(−)-menthol	doxorubicin menthoxycarbonylacetylhydrazone	cervix (KB-V1/Vbl)	IC_50_ = 17.8 ± 3.8 μM (24 h) IC_50_ = 18.4 ± 3.0 μM (48 h) IC_50_ = 10.3 ± 2.1 μM (72 h)	[84]
**47b**	(−)-menthol	doxorubicin 5-(menthoxycarbonyl)pentanoylhydrazone 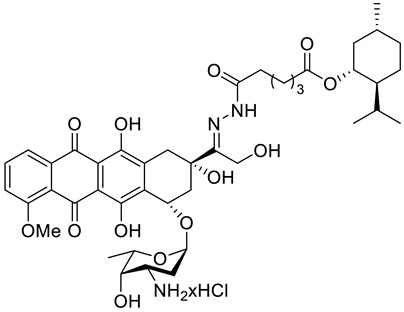	leukemia (HL-60)	IC_50_ = 0.39 ± 0.18 μM (24 h) IC_50_ = 0.33 ± 0.11 μM (48 h) IC_50_ = 0.33 ± 0.05 μM (72 h)	[84]
**47b**	(−)-menthol	doxorubicin 5-(menthoxycarbonyl)pentanoylhydrazone	melanoma (518A2)	IC_50_ = 1.2 ± 0.3 μM (24 h) IC_50_ = 0.42 ± 0.08 μM (48 h) IC_50_ = 0.32 ± 0.12 μM (72 h)	[84]
**47b**	(−)-menthol	doxorubicin 5-(menthoxycarbonyl)pentanoylhydrazone	breast (MCF-7/Topo)	IC_50_ = 6.7 ± 0.9 μM (24 h) IC_50_ = 4.7 ± 1.2 μM (48 h) IC_50_ = 2.4 ± 1.0 μM (72 h)	[84]
**47b**	(−)-menthol	doxorubicin 5-(menthoxycarbonyl)pentanoylhydrazone	cervix (KB-V1/Vbl)	IC_50_ > 100 μM (24 h) IC_50_ = 34.9 ± 15.7 μM (48 h) IC_50_ = 23.3 ± 12.5 μM (72 h)	[84]
**47c**	(−)-menthol	doxorubicin 8-(menthoxycarbonyl)octanoylhydrazone 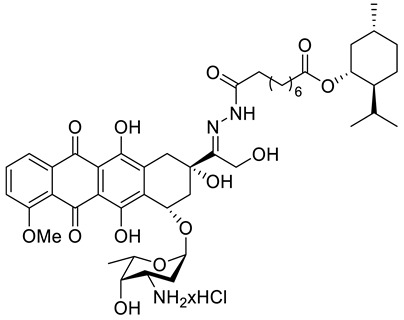	leukemia (HL-60)	IC_50_ = 0.40 ± 0.22 μM (24 h) IC_50_ = 0.49 ± 0.17 μM (48 h) IC_50_ = 0.37 ± 0.25 μM (72 h)	[84]
**47c**	(−)-menthol	doxorubicin 8-(menthoxycarbonyl)octanoylhydrazone	melanoma (518A2)	IC_50_ = 0.82 ± 0.20 μM (24 h) IC_50_ = 0.51 ± 0.17 μM (48 h) IC_50_ = 0.46 ± 0.12 μM (72 h)	[84]
**47c**	(−)-menthol	doxorubicin 8-(menthoxycarbonyl)octanoylhydrazone	breast (MCF-7/Topo)	IC_50_ = 10.2 ± 2.8 μM (24 h) IC_50_ = 7.2 ± 1.8 μM (48 h) IC_50_ = 4.3 ± 1.9 μM (72 h)	[84]
**47c**	(−)-menthol	doxorubicin 8-(menthoxycarbonyl)octanoylhydrazone	cervix (KB-V1/Vbl)	IC_50_ = 79.6 ± 6.8 μM (24 h) IC_50_ = 21.5 ± 3.7 μM (48 h) IC_50_ = 21.8 ± 3.8 μM (72 h)	[84]
**47d**	(−)-menthol	doxorubicin 11-(menthoxycarbonyl)undecanoylhydrazone 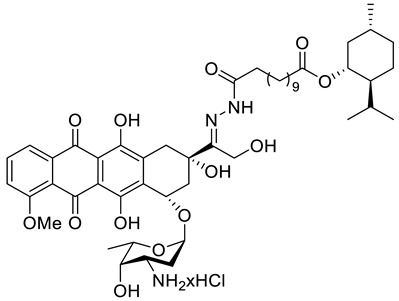	leukemia (HL-60)	IC_50_ = 0.30 ± 0.10 μM (24 h) IC_50_ = 0.25 ± 0.19 μM (48 h) IC_50_ = 0.11 ± 0.05 μM (72 h)	[84]
**47d**	(−)-menthol	doxorubicin 11-(menthoxycarbonyl)undecanoylhydrazone	melanoma (518A2)	IC_50_ = 0.23 ± 0.06 μM (24 h) IC_50_ = 0.16 ± 0.05 μM (48 h) IC_50_ = 0.06 ± 0.01 μM (72 h)	[84]
**47d**	(−)-menthol	doxorubicin 11-(menthoxycarbonyl)undecanoylhydrazone	breast (MCF-7/Topo)	IC_50_ = 7.1 ± 1.8 μM (24 h) IC_50_ = 2.6 ± 1.1 μM (48 h) IC_50_ = 2.6 ± 1.1 μM (72 h)	[84]
**47d**	(−)-menthol	doxorubicin 11-(menthoxycarbonyl)undecanoylhydrazone	cervix (KB-V1/Vbl)	IC_50_ = 30.5 ± 4.6 μM (24 h) IC_50_ = 17.1 ± 2.5 μM (48 h) IC_50_ = 8.8 ± 0.7 μM (72 h)	[84]
**48a**	(−)-menthol	(−)-menthyl[6-(aminomethyl)nicotinate]dichloridoplatinum(II) 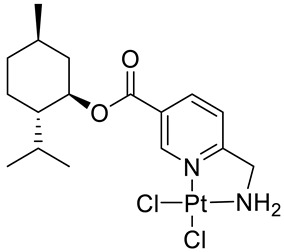	melanoma (518A2)	IC_50_ = 7.4 ± 0.1 μM (24 h)	[85,90]
**48a**	(−)-menthol	(−)-menthyl[6-(aminomethyl)nicotinate]dichloridoplatinum(II)	leukemia (HL-60)	IC_50_ = 8.0 ± 1.0 μM (24 h)	[85,90]
**48b**	(−)-menthol	(−)-menthyl derivative [6-(aminomethyl)nicotinate]dichloridoplatinum(II) 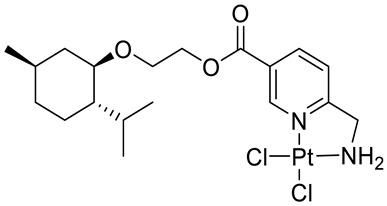	melanoma (518A2)	IC_50_ ≥ 50 μM (24 h)	[85]
**48b**	(−)-menthol	(−)-menthyl derivative [6-(aminomethyl)nicotinate]dichloridoplatinum(II)	leukemia (HL-60)	IC_50_ = n.d. (24 h)	[85]
**48c**	(−)-menthol	(−)-menthyl derivative [6-(aminomethyl)nicotinate]dichloridoplatinum(II) 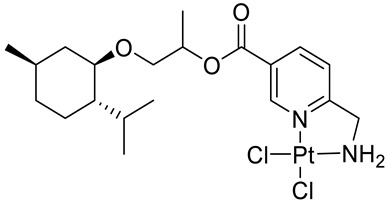	melanoma (518A2)	IC_50_ = 3.0 ± 2.4 μM (24 h)	[85]
**48c**	(−)-menthol	(−)-menthyl derivative [6-(aminomethyl)nicotinate]dichloridoplatinum(II)	leukemia (HL-60)	IC_50_ = 5 μM (24 h)	[85]
**48d**	(−)-menthol	(−)-menthyl derivative [6-(aminomethyl)nicotinate]dichloridoplatinum(II) 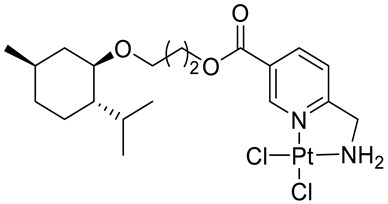	melanoma (518A2)	IC_50_ = 10.0 ± 5.6 μM (24 h)	[85]
**48d**	(−)-menthol	(−)-menthyl derivative [6-(aminomethyl)nicotinate]dichloridoplatinum(II)	leukemia (HL-60)	IC_50_ = 5.75 ± 1.8 μM (24 h)	[85]
**48e**	(−)-menthol	(−)-menthyl derivative [6-(aminomethyl)nicotinate]dichloridoplatinum(II) 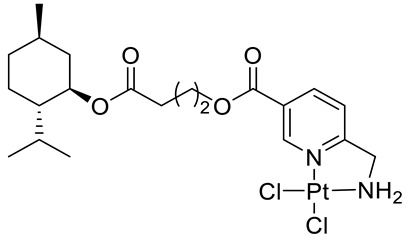	melanoma (518A2)	IC_50_ = 11.7 ± 4.0 μM (24 h)	[85]
**48e**	(−)-menthol	(−)-menthyl derivative [6-(aminomethyl)nicotinate]dichloridoplatinum(II)	leukemia (HL-60)	IC_50_ = 7 μM (24 h)	[85]
**48f**	(−)-menthol	(−)-menthyl derivative [6-(aminomethyl)nicotinate]dichloridoplatinum(II) 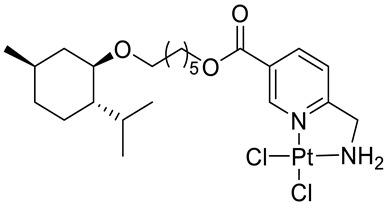	melanoma (518A2)	IC_50_ = 34 μM (24 h)	[85]
**48f**	(−)-menthol	(−)-menthyl derivative [6-(aminomethyl)nicotinate]dichloridoplatinum(II)	leukemia (HL-60)	IC_50_ = n.d. (24 h)	[85]
**48g**	(−)-menthol	(−)-menthyl derivative [6-(aminomethyl)nicotinate]dichloridoplatinum(II) 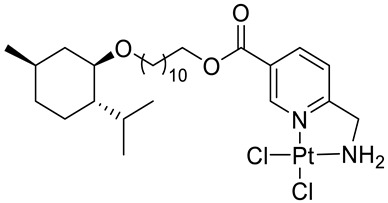	melanoma (518A2)	IC_50_ ≥ 50 μM (24 h)	[85]
**48g**	(−)-menthol	(−)-menthyl derivative [6-(aminomethyl)nicotinate]dichloridoplatinum(II)	leukemia (HL-60)	IC_50_ = n.d. (24 h)	[85]
**48h**	(+)-menthol	(+)-menthyl[6-(aminomethyl)nicotinate]dichloridoplatinum(II) 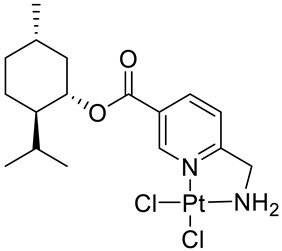	melanoma (518A2)	IC_50_ = 15.5 ± 0.9 μM (24 h)	[85,90]
**48h**	(+)-menthol	(+)-menthyl[6-(aminomethyl)nicotinate]dichloridoplatinum(II)	leukemia (HL-60)	IC_50_ = 7.0 ± 0.2 μM (24 h)	[85,90]
**48i**	(+)-menthol	(+)-menthyl derivative [6-(aminomethyl)nicotinate]dichloridoplatinum(II) 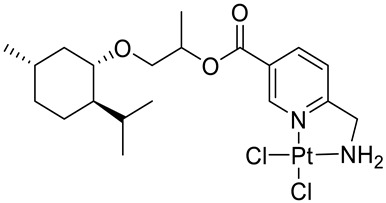	melanoma (518A2)	IC_50_ = 5.0 ± 2.0 μM (24 h)	[85]
**48i**	(+)-menthol	(+)-menthyl derivative [6-(aminomethyl)nicotinate]dichloridoplatinum(II)	leukemia (HL-60)	IC_50_ = 15 μM (24 h)	[85]
**48j**	(+)-menthol	(+)-menthyl derivative [6-(aminomethyl)nicotinate]dichloridoplatinum(II) 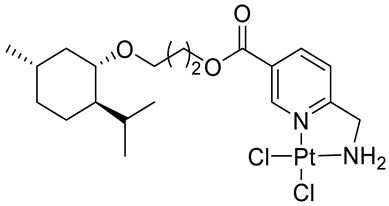	melanoma (518A2)	IC_50_ = 5.0 ± 1.4 μM (24 h)	[85]
**48j**	(+)-menthol	(+)-menthyl derivative [6-(aminomethyl)nicotinate]dichloridoplatinum(II)	leukemia (HL-60)	IC_50_ = 13 μM (24 h)	[85]
**48k**	(+)-neomenthol	(+)-neomenthyl[6-(aminomethyl)nicotinate]dichloridoplatinum(II) 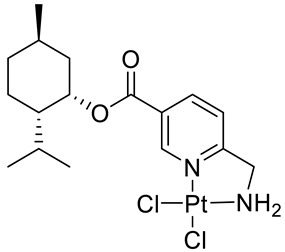	melanoma (518A2)	IC_50_ = 8.3 ± 0.8 μM (24 h)	[85,90]
**48k**	(+)-neomenthol	(+)-neomenthyl[6-(aminomethyl)nicotinate]dichloridoplatinum(II)	leukemia (HL-60)	IC_50_ = 9.8 ± 3.2 μM (24 h)	[85,90]
**48l**	(+)-neomenthol	(+)-neomenthyl derivative [6-(aminomethyl)nicotinate]dichloridoplatinum(II) 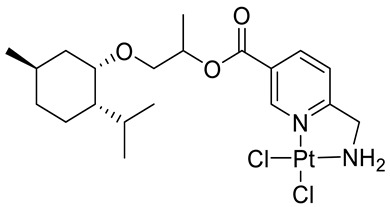	melanoma (518A2)	IC_50_ = 2.7 ± 0.35 μM (24 h)	[85]
**48l**	(+)-neomenthol	(+)-neomenthyl derivative [6-(aminomethyl)nicotinate]dichloridoplatinum(II)	leukemia (HL-60)	IC_50_ = 14 μM (24 h)	[85]
**48m**	(+)-neomenthol	(+)-neomenthyl derivative [6-(aminomethyl)nicotinate]dichloridoplatinum(II) 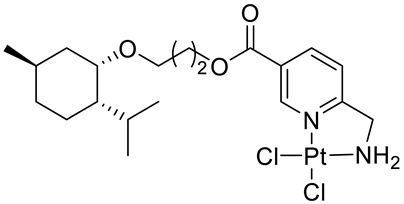	melanoma (518A2)	IC_50_ = 5.0 ± 1.2 μM (24 h)	[85]
**48m**	(+)-neomenthol	(+)-neomenthyl derivative [6-(aminomethyl)nicotinate]dichloridoplatinum(II)	leukemia (HL-60)	IC_50_ = 14 μM (24 h)	[85]
**48n**	(−)-neomenthol	(−)-neomenthyl[6-(aminomethyl)nicotinate]dichloridoplatinum(II) 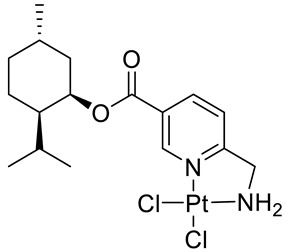	melanoma (518A2)	IC_50_ = 12.0 ± 7.1 μM (24 h)	[85]
**48n**	(−)-neomenthol	(−)-neomenthyl[6-(aminomethyl)nicotinate]dichloridoplatinum(II)	leukemia (HL-60)	IC_50_ = n.d. (24 h)	[85]

n.d. = not determined.

## 8. Therapeutic Deep Eutectic Solvents

Since Abbott et al. first introduced deep eutectic solvents (DESs) in 2003 [91], extensive research efforts have been dedicated to exploring the potential of these systems due to their desirable properties. DESs are mixtures of two or more compounds that, at a specific molar ratio, have a lower melting point than their individual components. This decrease is mainly attributed to hydrogen bond interactions between the different components in a DES [92]. The widespread interest in these systems is due to their many potential applications including gas separation and capture, biocatalysis and organic chemistry, power systems and battery technology, polymer fabrication, biomass processing, nanomaterial synthesis, pharmaceutical and medical research [15,16].

When DESs are synthesized from natural compounds, generally primary or secondary metabolites, they are termed natural deep eutectic systems (NADESs). A key benefit of using DESs is the possibility of a large array of different combinations, since not only can the substances that create such a system be modified, but so can their molar ratios. Therefore, these systems can be designed on a large scale at low cost and toxicity with special features that exhibit application-specific characteristics and desired activities. When one of the system components is an APIs or a bioactive compound, the resultant compound is referred to as a therapeutic deep eutectic system (THEDES) [93]. THEDESs have become more attractive in the pharmaceutical field due to their ability to increase APIs’ bioavailability. A popular monoterpene, menthol, is one of the most common hydrogen bond acceptors (HBAs) among the THEDESs [94] and has already been combined with a large variety of APIs (e.g., ibuprofen, lidocaine, fluconazole and captopril) to create such eutectic systems [93].

Rodrigues et al. [95] prepared NADESs by (i) mixing various terpenes and also (ii) mixing various terpenes with saturated fatty acid (Table 2) as follows: (*S*)-(−)-perillyl alcohol **20** (POH), (±)-menthol (ME) and myristic acid (MA) were chosen as molecules that might donate a hydrogen bond through their hydroxyl group, while (*S*)-(−)-perillyl alcohol (POH), (±)-camphor (CAM), (±)-menthol (ME), eucalyptol (EU) and myristic acid (MA) might act as HBAs through their oxygen atoms.

NADESs showed higher cytotoxicity than their corresponding physical mixtures, except for ME:MA eutectic system, prepared at an 8:1 ratio, compared with (±)-menthol and myristic acid physical mixture (PM). The antiproliferative effects of all of the NADESs were stronger in HT-29 cells than the physical mixtures of the tested compounds. Hence, the 8:1 ME:MA ratio seems to be the most promising NADESs, since it exhibited a stronger antiproliferative effect with less cytotoxicity than its physical counterpart (Table 3).

Another group of researchers investigated a THEDES consisting of (*S*)-(−)-perillyl alcohol **20** (Figure 9) and ibuprofen (IBU) at different molar ratios in their study of THEDES cytotoxicity against colorectal cancer in vitro. The main purpose of their work was to develop a THEDES by bringing together the natural monoterpene POH and the active pharmaceutical ingredient in IBU to increase their solubility and bioavailability as a novel therapeutic formulation. As a control for the THEDES, a physical mixture of POH–IBU was analyzed in parallel and yielded antiproliferative properties with an EC_50_ of 4.43–4.51 mM. Concerning the anticancer properties of these newly prepared THEDES, the EC_50_ outcome from a cytotoxicity analysis of both eutectic systems showed an increase in the cytotoxicity against healthy colonic cells due to the increased POH ratio in eutectic. The therapeutic properties of the POH:IBU eutectic system designed in this study depend on the molar ratio. Different molar ratios may lead to selective treatments of POH:IBU. Though both molar ratio systems exhibited similar anticancer activity, POH:IBU (3:1) inhibited colorectal cancer cells more effectively with cytotoxic and antiproliferative efficacy at 8.46 and 1.316 mM, respectively. Furthermore, POH:IBU (3:1) had no apparent impact on healthy cell viability and presented a more promising selectivity index (5.89). The 8:1 molar ratio of POH:IBU exhibited cytotoxic and antiproliferative effects, respectively at 4.35 and 1.37 mM with selectivity index 2.60. Therefore, both systems presented interesting activity for the anti-proliferation assay, and a POH:IBU ratio of 3:1 appears to be the most promising for further development as a potential drug component [96].

Despite the promising studies detailing limonene’s chemotherapeutic effects against multiple cancer cell lines (e.g., lung, breast, prostate and gastrointestinal), it is also highly toxic to healthy cells. Because this terpene is not selective for cancer cells, it is not a viable therapeutic candidate on its own [97]. Therefore, it follows to test systems that combine limonene with otherwise selective agents. Pereira et al. [97] investigated the capacity of several limonene-based THEDES to treat cancer. They formulated 14 THEDESs by mixing saturated fatty acids and menthol or IBU with (*S*)-limonene (Table 4).

Their results indicate that all of the tested THEDESs present antiproliferative properties (Table 5); however, only IBU:LIM at a 1:4 ratio successfully inhibits HT29 cell proliferation without comprising viability. Furthermore, their results suggest that the mechanism of action of LIM:IBU (1:4) is different than ibuprofen or limonene alone, which speaks to the synergistic effect of DESs. The authors tuned the selectivity of (*S*)-limonene towards the HT29 cell line without compromising the viability of the healthy cells, thereby actualizing the potential of a proper eutectic system containing this monoterpene. Furthermore, pairing limonene with IBU enhances the anti-inflammatory activity of IBU, which is likely an essential component of anticancer therapies.

## 9. Conclusions

Monoterpenes and their derivatives are crucial to the design and synthesis of new biologically active compounds, including pharmaceutical agents. This review focuses on the data describing the various antitumor properties exhibited by selected monoterpenes including carvone, carvacrol, perillyl alcohol, perillaldehyde, limonene, menthol and their derivatives.

The key strategy used in drug discovery and development is functional group modification and/or the implementation of specific moieties. Interestingly, some derivatives might be more effective than their unsubstituted counterparts. A good example of this phenomenon is (−)-perillaldehyde 8,9-epoxide, which showed excellent cytotoxicity compared with (−)-perillaldehyde (GI = 96.32–99.89% versus GI = 59.28–83.03% respectively, against OVCAR-8, HCT-116 and SF-295 human tumor cell lines). Another example is the introduction of 4-(2-methoxyphenyl)piperazine to the carvone or limonene, which significantly increased their antiproliferative effects against human prostate cancer LNCaP cells. The presented literature shows that bioactivity against tumor cells of used monoterpenes is associated with their stereochemistry (e.g., the (+)-carvone and the opposite (–)-isomer exerted distinct effects on healthy and tumor cells).

It is worth mentioning that the available literature contains very little information on the selectivity of action on neoplastic and healthy cells. Moreover, there is a lack of tests for original terpenes to compare their anticancer effect with those of the obtained derivatives.

This review highlights the importance of novel approaches to delivering monoterpenes that address issues such as poor solubility, low bioavailability or high volatility, etc. Nanoparticles and DESs are examples of such systems that can optimize the biological activity of these promising compounds. Moreover, THEDESs have opened possibilities by combining natural monoterpenes with known pharmaceutical agents to create a novel potential therapeutic formulation far more potent than its components alone. In the case of loading of (+)-limonene 1,2-epoxide into SLNs result in cell viability of 76.27 ± 1.63% (10 µg/mL for 48 h), which may indicate a low toxic effect on the non-tumor cell line HaCaT relative to the pure unloaded (+)-limonene. Regarding the antiproliferative effect, all presented NADESs demonstrated a stronger capability in inhibiting HT-29 cell proliferation than the physical mixture of compounds, which demonstrates the benefits of creating such eutectic systems.

There has been considerable progress in the use of monoterpenes and their derivatives towards the development of novel alternatives for the treatment and prevention of cancer, as shown by the increasing body of evidence in the literature from both academia and industry. These compounds and their related systems, such as DES, are excellent therapeutic candidates ready to play an important role in pharmacotherapy.

## Figures and Tables

**Table 2:** Natural deep eutectic systems containing various terpenes as powerful inhibitors of HT-29 cell proliferation.

Counterpart A	Counterpart B	Molar Ratio	Abbreviation
Perillyl alcohol	Camphor	1:1	PA:CA (1:1)
Menthol	Perillyl alcohol	1:1	ME:PA (1:1)
Menthol	Camphor	1:1	ME:CAM (1:1)
Menthol	Eucalyptol	1:1	ME:EU (1:1)
Menthol	Myristic acid	8:1	ME:MA (8:1)

**Table 3:** EC_50_ values (mg/mL) for individual components, NADESs and physical mixtures in Caco-2 and HT-29 Cells, after an incubation period of 24 h.

Samples	Cytotoxicity (Caco-2 Cells)	Antiproliferative Effect (HT-29 Cells)
NADESs		
PA:CA (1:1)	0.89 ± 0.20	0.75 ± 0.36
ME:PA (1:1)	0.91 ± 0.08	0.57 ± 0.02
ME:CAM (1:1)	1.26 ± 0.02	1.54 ± 0.24
ME:EU (1:1)	1.58 ± 0.08	1.21 ± 0.07
ME:MA (8:1)	3.67 ± 0.34	0.84 ± 0.18
Individual components		
PA	0.74 ± 0.24	0.36 ± 0.03
CAM	>5.00	>5.00
ME	1.68 ± 0.50	2.67 ± 1.28
EU	>5.00	3.09 ± 0.24
MA	>1.50	>1.50
Physical mixtures (PM)		
PM—PA:CA (1:1)	2.45 ± 0.29	3.82 ± 0.37
PM—ME:PA (1:1)	1.34 ± 0.11	1.61 ± 0.38
PM—ME:CAM (1:1)	3.63 ± 0.21	3.08
PM—ME:EU (1:1)	3.31 ± 0.34	2.95 ± 1.31
PM—ME:MA (8:1)	0.72	5.42 ± 1.58

**Table 4:** Limonene-based therapeutic deep eutectic solvents.

Counterpart A	Counterpart B	Molar Ratio	Abbreviation
Myristic acid	Limonene	1:1	MA:LIM
Myristic acid	Limonene	1:2	MA:LIM
Myristic acid	Limonene	2:1	MA:LIM
Capric acid	Limonene	1:1	CAP:LIM
Capric acid	Limonene	1:2	CAP:LIM
Capric acid	Limonene	2:1	CAP:LIM
Menthol	Limonene	1:1	ME:LIM
Menthol	Limonene	1:2	ME:LIM
Menthol	Limonene	2:1	ME:LIM
Ibuprofen	Limonene	1:1	IBU:LIM
Ibuprofen	Limonene	1:2	IBU:LIM
Ibuprofen	Limonene	2:1	IBU:LIM
Ibuprofen	Limonene	1:4	IBU:LIM
Ibuprofen	Limonene	1:8	IBU:LIM

**Table 5:** EC_50_ values (mM) for individual components and THEDESs in Caco-2 and HT-29 Cells, after an incubation period of 24 h

Samples	Cytotoxicity (Caco-2 Cells)	Antiproliferative Effect (HT-29 Cells)
THEDESs		
CAP:LIM (1:1)	0.918 ± 0.042	0.6901 ± 0.105
ME:LIM (1:1)	2.314 ± 0.421	0.8023 ± 0.016
IBU:LIM (1:4)	10.50 ± 0.883	2.390 ± 2.919
IBU:LIM (1:8)	3.323 ± 0.228	1.137 ± 0.055
Individual components		
IBU	2.893 ± 0.059	2.346 ± 0.088
CAP	1.334 ± 0.223	0.341 ± 0.081
LIM	2.638 ± 0.108	0.661 ± 0.025
ME	8.078 ± 0.810	4.730 ± 16.14

## Abbreviations

ADR	Adriamycin
APIs	Active pharmaceutical ingredient
AML	Acute myeloid leukemia
AMPK	5′ Adenosine monophosphate-activated protein kinase
APIs	Active pharmaceutical ingredients
BW	Body weight
BBB	Blood–brain barrier
CAM	Camphor
CAP	Capric acid
CV	Carvacrol
CVN	Carvone
CoN	Colon epithelial cells
COX-2	Cyclooxygenase-2
cSLNs	Cationic solid lipid nanoparticles
DEN	Diethylnitrosamine
DESs	Deep eutectic solvents
DMBA	7,12-Dimethylbenz[a]anthracene
DMH	1,2-Dimethylhydrazine
EC_50_	Effective concentration of a drug or concentration of a drug which is calculated to inhibit virus induced cell death by 50%
ERK	Extracellular signal-regulated kinases
EU	Eucalyptol
GBA	Glioblastoma
GI_50_	The 50% growth inhibition percentage
GI%	The cell growth inhibition percentage values
HBAs	Hydrogen bond acceptors
HC	Hepatocellular carcinoma
HDFs	Human dermal fibroblasts
HRPC	Hormone-refractory prostate cancer
IBU	Ibuprofen
IC_50_	Concentration of an inhibitor where the response (or binding) is reduced by half
LIM	limonene
LIM-SLNs	Solid lipid nanoparticles formulation with (+)-limonene 1,2-epoxide and glycerol monostearate
LPO	Lipid peroxidation
MAPK	Mitogen-activated protein kinase
MA	Myristic acid
ME	Menthol
MTT	3-(4,5-Dimethylthiazol-2-yl)-2,5-diphenyltetrazolium bromide
MTT assay	The colorimetric assay based on the reduction of yellow tetrazolium salt (MTT) to purple formazan
NADESs	Natural deep eutectic systems
NEO100	Highly pure perillyl alcohol
NR	3-Amino-7-dimethyl-2-methylphenazine hydrochloride
NR assay	The neutral red (NR) dye uptake assay
NEO212	Compound built of perillyl alcohol and temozolomide
p21^waf1^	Cyclin-dependent kinase inhibitor
PAH	Perillaldehyde
3-PCA	Pyrrolidine-3-carboxylic acid
PE	Perillaldehyde 1,2-epoxide
PLK1	Polo-like kinase 1
PM	Physical mixtures
POH	Perillyl alcohol
ROS	Reactive oxygen species
SLNs	Solid lipid nanoparticles
SRB	Sulforhodamine B
SAR	Structure-activity relationship
THEDES	Therapeutic deep eutectic system
THEDESs	Therapeutic deep eutectic solvents
TMZ	Temozolomide
TRPM8	Transient receptor potential melastatin member 8
